# Lossen Rearrangement of p-Toluenesulfonates of N-Oxyimides in Basic Condition, Theoretical Study, and Molecular Docking

**DOI:** 10.3389/fchem.2021.662533

**Published:** 2021-04-15

**Authors:** Monika Kijewska, Abeer A. Sharfalddin, Łukasz Jaremko, Marta Cal, Bartosz Setner, Miłosz Siczek, Piotr Stefanowicz, Mostafa A. Hussien, Abdul-Hamid Emwas, Mariusz Jaremko

**Affiliations:** ^1^Faculty of Chemistry, University of Wrocław, Wrocław, Poland; ^2^Department of Chemistry, Faculty of Sciences, King Abdulaziz University, Jeddah, Saudi Arabia; ^3^Division of Biological and Environmental Sciences and Engineering, King Abdullah University of Science and Technology, Jeddah, Saudi Arabia; ^4^Department of Chemistry Faculty of Science, Port Said University, Port Said, Egypt; ^5^Core Labs, King Abdullah University of Science and Technology (KAUST), Jeddah, Saudi Arabia

**Keywords:** p-toluenesulfonates, N-oxyimides, XRD, NMR, DFT, molecular docking

## Abstract

The sulfonic esters of N-oxyimides are a group of compounds with a wide range of biological activities, as well as a unique reactivity toward amines. They undergo this reaction with primary amines and other nucleophilic reagents according to a Lossen-like rearrangement. The reaction is initiated by nucleophilic attack on a carbonyl group in the succinimide ring followed by isocyanate formation, which next interacts with another nucleophile molecule forming an addition product (e.g., ureido or urethane derivative). However, the secondary amines are also susceptible to other reactions leading to products containing the maleimide ring formed by sulphonic acid elimination. In the case of tertiary amines, this reaction is predominant. To explain the phenomenon of the reactivity of the N- oxyimides toward different types of amines, we employed various spectroscopic and X-ray approaches as well as DFT calculation. Results suggest that the basicity of the amine used for the reaction plays a crucial role in the reaction mechanism that eventually dominates the entire chemical process. Moreover, we applied molecular docking to investigate the ability of the products to act as serine protease inhibitors using human leukocyte elastase (HLE).

## Introduction

Non-protein amino acids play many essential roles in the biochemical pathways of living organisms. Identifying new non-protein amino acid derivatives can facilitate new important applications for this group of compounds, e.g., in medicine, as well as facilitating pathway investigations. Product diversity is caused by different mechanisms of contemporary nucleophile attack. The sulfonic esters of N-oxyimides belong to a group of chemical compounds that is characterized by specific chemical and biological properties. It has been shown that sulfonic esters of N-oxyimides, in contrast to the carboxylic esters of the Noxyimides, react with the nucleophiles like amines through the carbonyl groups of the succinimidic ring to form ureido derivatives. The mechanism of these reactions, which is related with the Lossen rearrangement, has been previously described (Bauer and Exner, 1974; Groutas et al., 1986; Ranganathan et al., 1997). Moreover, many of these compounds are known for their inhibitory properties against serine proteases, mainly human leukocyte elastase (HLE) (Neumann and Gütschow, 1994; Abell and Oldham, 1999) and anti-fungial properties (Duraipandiyan and Ignacimuthu, 2011; Lamberth, 2016). This enzyme is an object of clinical interest due to its involvement in pulmonary emphysema and other inflammatory ailments (Hunninghake and Gadek, 1981–1982; Tirouvanziam, 2006). There are also reports on the inhibition of other serine proteases, like chymotrypsin, and cathepsin G (Groutas et al., 1989a).

The sulfonic esters of N-hydroxyimides with the reaction of the primary amines as nucleophiles generate an isocyanate via an enzyme-induced Lossen-type rearrangement. According to the mechanism postulated by Groutas et al. (1989b), the isocyanate group acetylates a histidine. In review moiety in the active center inactivating the enzyme rapidly and irreversibly. The molecular bases of reactivity of sulphonic esters of N-oxyimides toward nucleophiles and serine proteases (most likely the Ser195 residue) (Groutas et al., 1989b) have seemingly a lot in common with the rearrangement mentioned above, but the details still remain unknown. It was also shown in one of our previous studies that sulfonic esters of N-hydroxyimides react with the weaker nucleophiles, like primary alcohols (to which the reaction is limited), yielding as a result of the Lossen rearrangement in N(α)-urethane-protected β- and γ-amino acid derivatives in the simple one-pot synthesis (Cal et al., 2013).

A recent attempt was made to explain this biochemical phenomenon of sulfonic esters of N-oxyimides. X-ray crystallography was applied to some sulfonic esters of N-oxyimides, which form stable single crystals suitable for X-ray measurements (Stefanowicz et al., 2005, 2006). Detailed analysis of the investigated compounds geometry in the crystalline state shows a pyramidal shape geometry of the succinimidic ring nitrogen atom, which is the unique geometrical feature common to all investigated compounds that was observed only for the previously reported crystal structure of another sulfonic ester of Noxyimide, N-benzenesulfonyloxynaphthalimide (Grigorieva and Chetkina, 1977). The nitrogen atom displacement from the plane defined by the three adjacent atoms can vary from 0.150(1) Å for 1-[(2-naphthylsulfonyl)oxy]pyrrolidine-2,5-dione (Stefanowicz et al., 2005) and also Noxyimide N-benzenesulfonyloxynaphthalimide (Grigorieva and Chetkina, 1977) to 0.243(2) Å for 2-[(4-methylphenyl)sulfonyl]oxy-1H-isoindole-1,3(2H)-dione (Stefanowicz et al., 2006). Such a geometrical feature does not exist for the previously reported crystalline structures of carboxylic esters of the N- oxyimides (Crisma and Toniolo, 2002) and also for almost-planar N- hydroxyimides (Brown, 1961; Karolak-Wojciechowska et al., 1993; Miao et al., 1995; Jones, 2003; Stefanowicz et al., 2007) or slightly puckered N-halogensuccinimides (Wakefield and Wright, 1980).

The reason for this unique feature of the sulfonic ester has remained unknown. In particular, it is not known if the differences in the succinimide ring geometry result from the significantly different influence of the sulfonic and carboxylic groups on the electron density distribution in the ring, or if they come from intermolecular interactions in the solid phase and the influence of the groups is secondary. No investigations have been performed to elucidate the nitrogen atom geometry of the esters in solution.

To date, it is suspected that the pyramidal geometry of the nitrogen atom, which affects other geometrical parameters of the succinimidic ring, may play a pivotal role in explaining the sulfonic esters biochemical reactivity. The C-N bond lengths observed for all sulfonic esters of N-oxyimides in the solid state are longer than typical C-N peptide bonds and also longer than typical C-N bonds for the succinimide ring (Stefanowicz et al., 2005). These facts show that in the solid phase, the sulfonic esters of the N- oxyimides are characterized by the different electron density distribution of the succinimidic ring than other cyclic imides. Mentioned geometrical features, which possess the unique character are considered to result from the amide resonance reduction, which means that the shift of the electron pair is significantly limited, confirmed by longer C—N bond lengths. The electron pair shifting along the C—N bond length is considered to decrease the electron density on the carbonyl carbon atom, which becomes more susceptible to the nucleophile attack (1). The lack of any correlation between the molecular packing in the crystalline lattices and the nitrogen atom geometry of investigated esters has been also confirmed (1). It is worth mentioning that β- lactams, which are well-known for their biological properties, are also characterized by the pyramidal shape nitrogen atom. The nitrogen atom pyramidal geometry is believed to be responsible for the biological properties of this class of compounds (Cohen, 1983). However, others claim that the pyramidal geometry of the nitrogen atom is not the only source of these specific biological properties of β-lactams because of the lack of a clear and also simple correlation between this pyramidal geometry of the nitrogen atom investigated in the solid state and the strength of the antibacterial properties of this class of compounds (Gütschow, 1999).

## Results and Discussion

In contrast to the reaction of sulfonic ester of N-hydroxyl imide based on aspartic acid (1C) with the primary amines, the reaction of 1C with secondary amines predominantly yields the product containing the maleimide ring. This study is focused mainly on the explanation of this phenomenon with the use of DFT calculation approaches. In addition, we present and discuss also a crystal structure of one of the reaction products (noted as 2C2L) between the sulfonic ester of N-oxyimides and the primary amine.

**Scheme 1:**
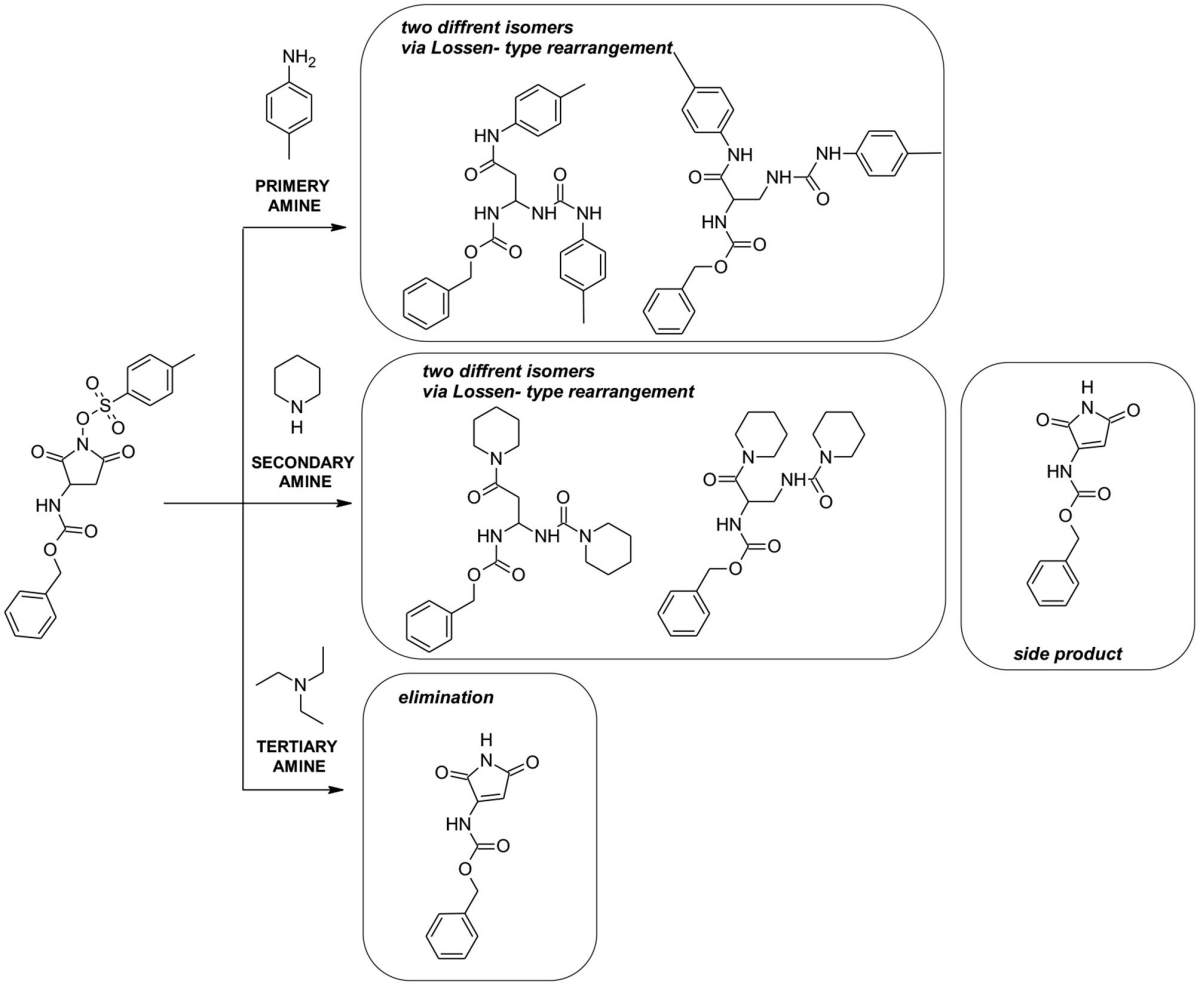
Reaction of sulfonic ester of N- hydroxyl imide based on aspartic acid with primary, secondary, and tertiary amines.

The reactions between the sulfonic ester of N- hydroxyl imide based on aspartic acid or glutamic acid with selected primary and secondary amines were examined resulting in the formation of products via a Lossen-type rearrangement (Scheme 1). Besides the main products after Lossen rearrangement for the secondary amine (e.g., piperidine), the formation of the byproduct, N-protected 3-amino-1H-pyrrole-2,5-dione, was observed. In contrast, the use of a tertiary amine in the reaction resulted in obtaining only one product, N-protected 3-amino-1Hpyrrole-2,5-dione. This phenomenon was explained by competition between basicity and nucleophilic properties. In the case of using a tertiary amine in the first step, the reaction mechanism involves deprotonation followed by the elimination of the Tosyl group, which was further confirmed by DFT calculations (Scheme 1). This new reaction product resulting in reaction of the sulfonic ester of N-hydroxyimide based on aspartic acid with triethylamine was described and characterized by NMR spectroscopy and X-ray crystallography techniques.

The reagents [cyclic hydroxyimide of N-protected aspartic acid (1C) or glutamic acid (2C)] were prepared by the procedure described in Supporting Information. In our experiments, we studied the reaction of compounds 1C and 2C with various primary (N-propylamine, toluidyne), secondary (piperidine, morpholine), and tertiary (triethylamine) amines in two solvents DMSO and THF at room temperature for 24 h and the corresponding products via Lossen-type rearrangement were obtained except triethylamine (Scheme 1). The results are summarized in Supplementary Table 1. The identity of the obtained products were confirmed by MS analysis (Supplementary Table 2). Representative ESI-MS spectra are presented in Supplementary Figure 1. The ESI-MS spectrum is dominated by the signal representing the charged ion [M+Na]^+^ corresponding to the compound 1C3L with sodium ion.

**Figure 1:**
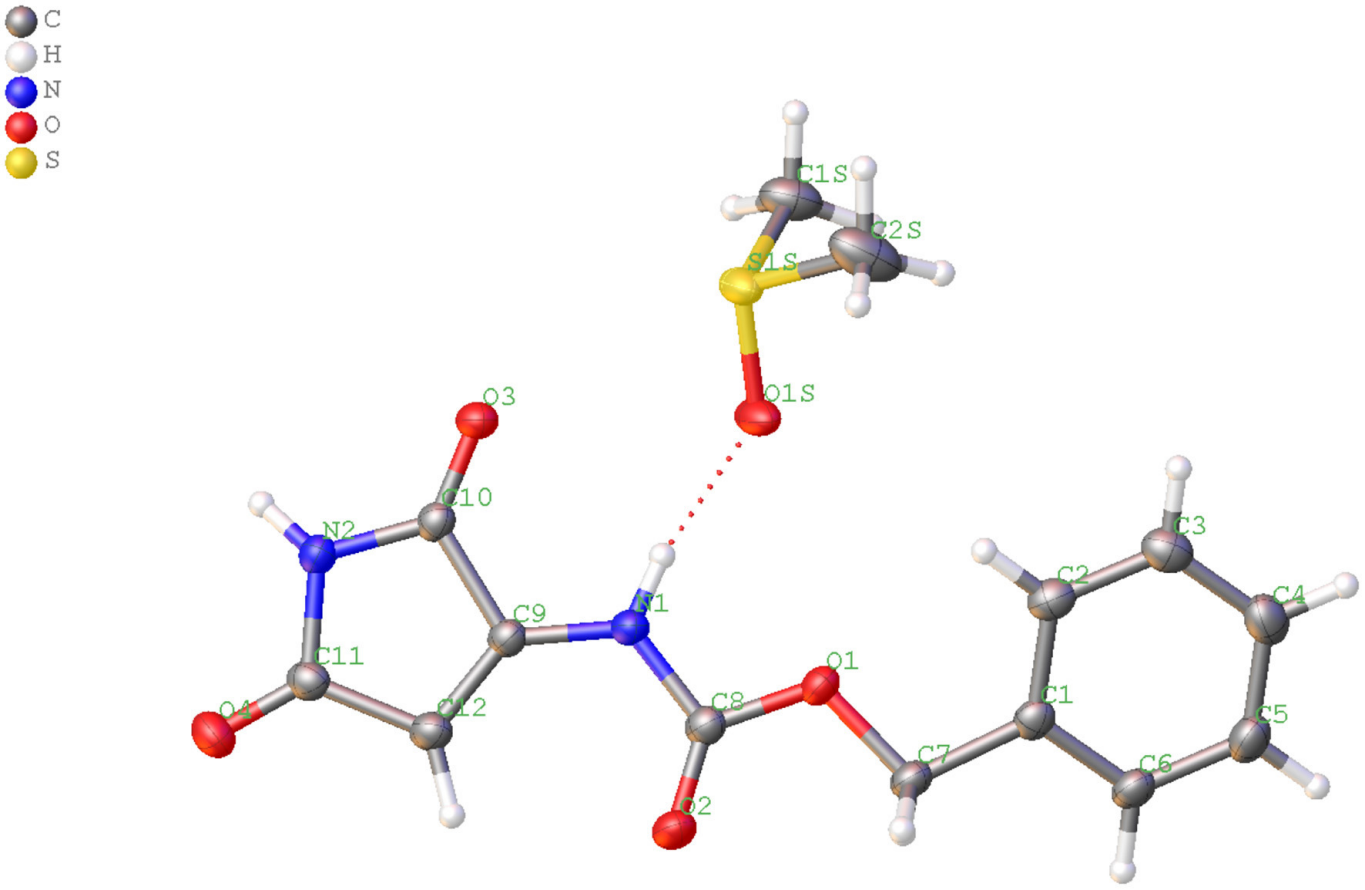
View of the independent molecules with atom-numbering of N-protected 3-amino-1H-pyrrole-2,5-dione and DMSO in the asymmetric unit.

**Figure 2:**
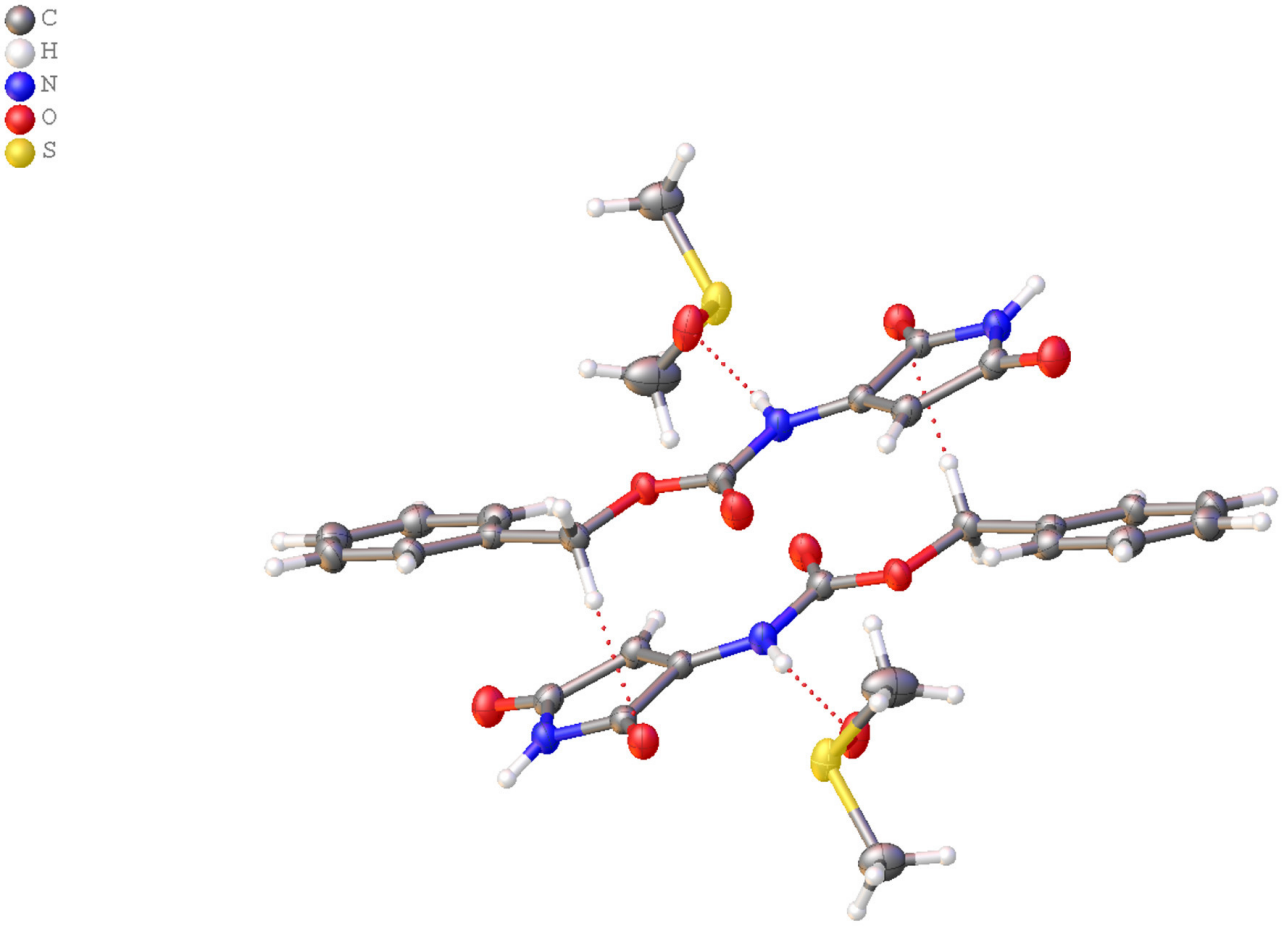
Arrangement of molecules and solvent DMSO.

MS/MS fragmentation confirmed the structure of all obtained compounds via Lossen-type rearrangement. Fragmentation pathways for all compounds, both p-toluenesulfonates of Noxyimide derivatives of glutamic and aspartic acid, are similar. These data are presented in the Supporting Information (Supplementary Figures 6–10). Unexpectedly in the reaction with secondary amine (piperidine), we found the formation of N-substituted 3-amino-1H-pyrrole-2,5-dione in very low yield (Scheme 1) that was further crystallized and analyzed by HR-MS (Supplementary Figure 2) and NMR (Supplementary Figures 12–15), and characterized by X-ray crystallography (Figures 1, 2 and Supplementary Figures 16, 17). The results are summarized in Supplementary Tables 3–7. According to the literature, this compound may form in photolytic reaction p-toluenesulphonic acid. Moreover, the amount of this compound that is formed depends on the nucleophilic properties of the used amine. For tertiary, the amine rearrangement product was not observed, and only benzyl (2,5-dioxo-2,5-dihydro-1H-pyrrol-3-yl) carbamate product was recorded (1C1L).

### Crystal Structure

The structure of compound 1C1L was confirmed by X-ray structure analysis. During recrystallization two kinds of crystals were obtained with (CCDC- 2064551 - **1C1L**·DMSO) and without (CCDC-2064552 - **1C1L**) solvent molecule but only first one will be further discussed. The experimental data and obtained results for both structure were placed in Supporting Information.

The compound **1C1L**·DMSO crystallizes in the monoclinic space group P2_1_/c. The independent molecules are shown in Figure 1. Crystallographic data and parameters are given in Supplementary Table 3. The N-protected 3-amino-1H-pyrrole-2,5-dione molecule adopt a distorted planar geometry with slightly twisted benzyl group, C6 - C1 - C7 - O1 dihedral angel of−160.50(18)° (Figure 2). The distance between atoms C9 – C12 is 1.339(3)Å, clearly indicates the presence of double bond (Allen et al., 1987).

The DMSO and N-protected 3-amino-1H-pyrrole-2,5-dione molecules are linked together through N—H?O hydrogen bonds (Figure 2 and Supplementary Table 4). In the crystal structure, aromatic π-π stacking interactions between benzyl and pyrrole groups are observed (centroid–centroid distances 3.4382(11) Å).

**Figure 3:**
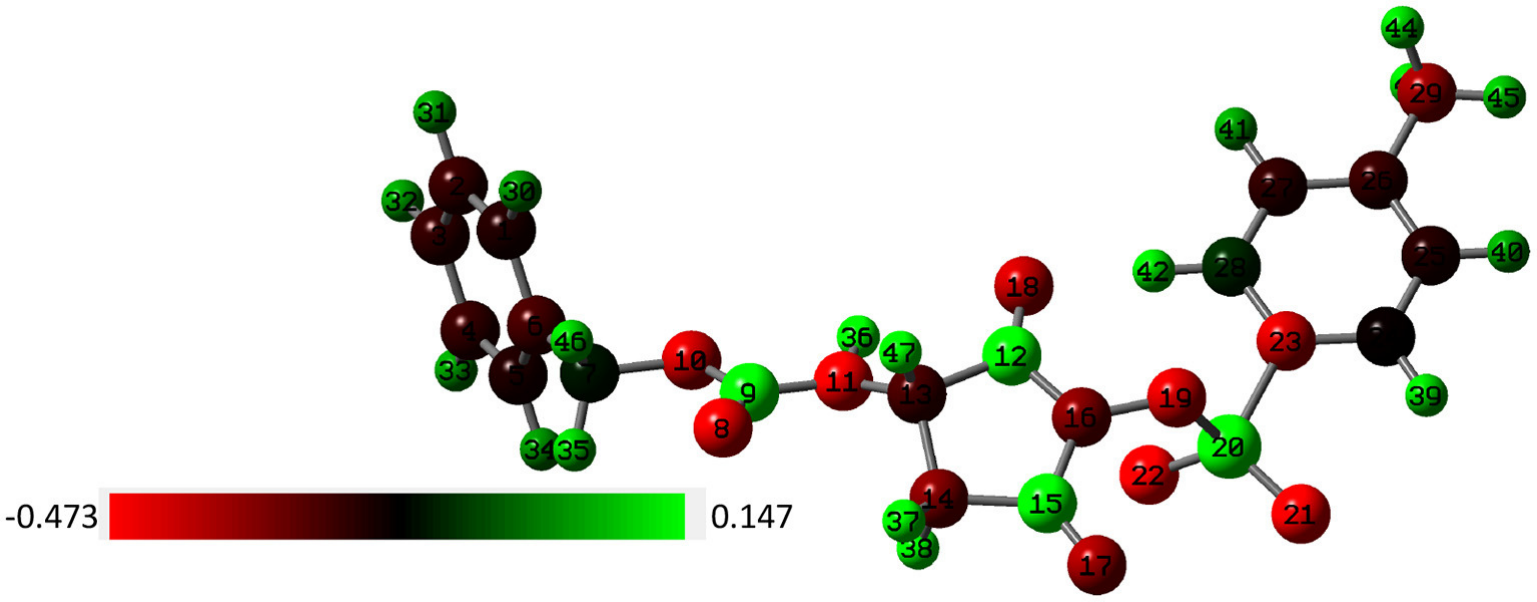
The charge dissolution over the p-toluenesulfonates of N-oxyimide and the atom number by NBO method (red color indicates to negative charge molecule while green color for the positive charge).

Experimental data demonstrate that formation of the 1H-pyrrole-2,5-dione derivative is possible only in a system with a succinimide ring substituted with a nitrogen atom. Arylosulphonates of N-hydroxysuccinimide are not susceptible to this reaction. To gain insight into the mechanism of the obtained minor piperidine product, we utilize the DFT approach to optimize the p-toluenesulfonates of N-oxyimide and investigate the active sites within the molecule. For this purpose, we utilized the signal crystal parameters for the p-toluenesulfonates of N-oxyimide in our previous work (Stefanowicz et al., 2006) to build the molecular and to create the input file. Figure 3 shows the optimized molecular structure with the atoms number and associated charge distribution.

**Figure 4:**
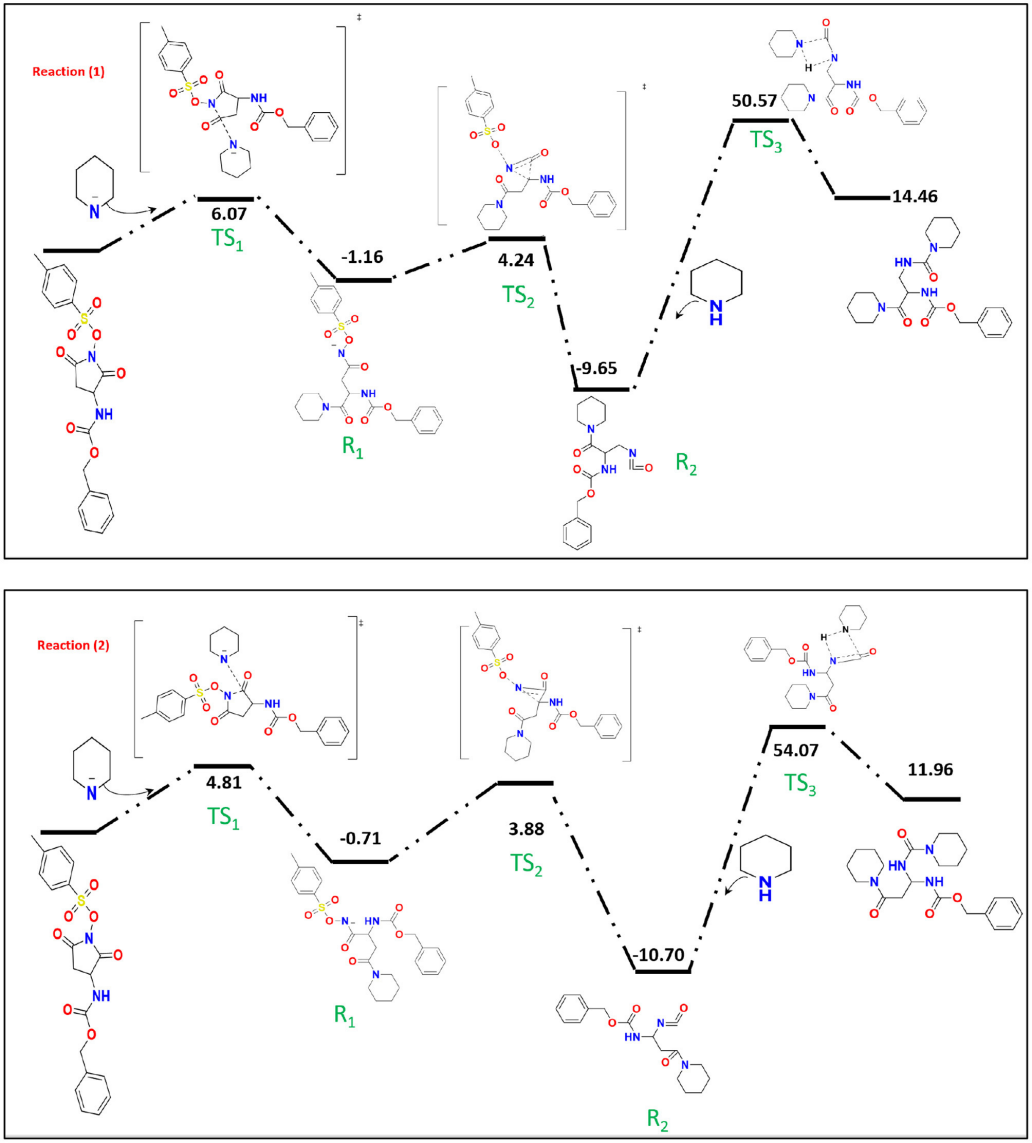
The proposal DFT reaction mechanisms for different path of the nucleophilic attack.

Mulliken population is usually combined with calculation in order to investigate the reactive sites within a molecule (Carbó-Dorca and Bultinck, 2004). Moreover, the natural atomic charge (NBO) is sensible approach when trying to attain some descriptors for molecule reactivity. The Mulliken charge distribution of the pyrrolidine-dione ring, Table 1, shows the C12 and C15 for the carbonyl groups have the highest positive charge among the ring atoms. Interestingly, the positive atomic charges indicated that the nucleophilic attack prefers to start with the C12 (0.452) than C15 (0.393). In contrast, the NBO values present the two carbon atoms are equally play as electrophilic sites in the ring. Despite both reaction paths are prospective, we performed the transition calculation for both conceivable paths of the secondary amine nucleophilic attack. Figure 4 shows the reaction mechanism of the amine nucleophilic attack by R1 and R2. The reaction pathway toward C15 (R1) via transition state has energy barrier of 6.07 kcal/mol to form the anionic intermediate. The Lossen rearrangement has energy barrier 5.41kcal/mol to form the stable molecule R2 ΔG = −9,65 Kcal/mol. Starting the second addition of the pieridine with energy barrier −40.92 kcal/mol to obtain the final product with 14.46 kcal/mol. On the other hand, the interaction with C12, R2, started with transition energy 4.81 kcal/mol to produce the anionic molecule. This was followed with arrangement with energy barrier 3.17 kcal/mol and formed R2. The final stage has an energy barrier 43.37 kcal/mol and formed a stable molecule (11.96 kcal/mol). Comparing the two paths, we could indicate that R2 is prefer than R1 with energy difference value 2.51 kcal/mol between the two products. Another observation that could be added, the steric hindrance effects from the two hydrogens connected to C15 tended to slow the reaction and give another proof of this preference.

**Table 1:** The atoms charge for pyrrolidine-dione ring calculated by Muliken and NBO.

**Atom**	**C12**	**C13**	**C14**	**C15**	**N16**	**H37**	**H38**	**H47**
Mulliken	0.452	−0.119	−0.217	0.393	−0.226	0.172	0.173	0.177
NBO	0.707	−0.087	−0.478	0.708	−0.183	0.244	0.245	0.226

**Figure 5:**
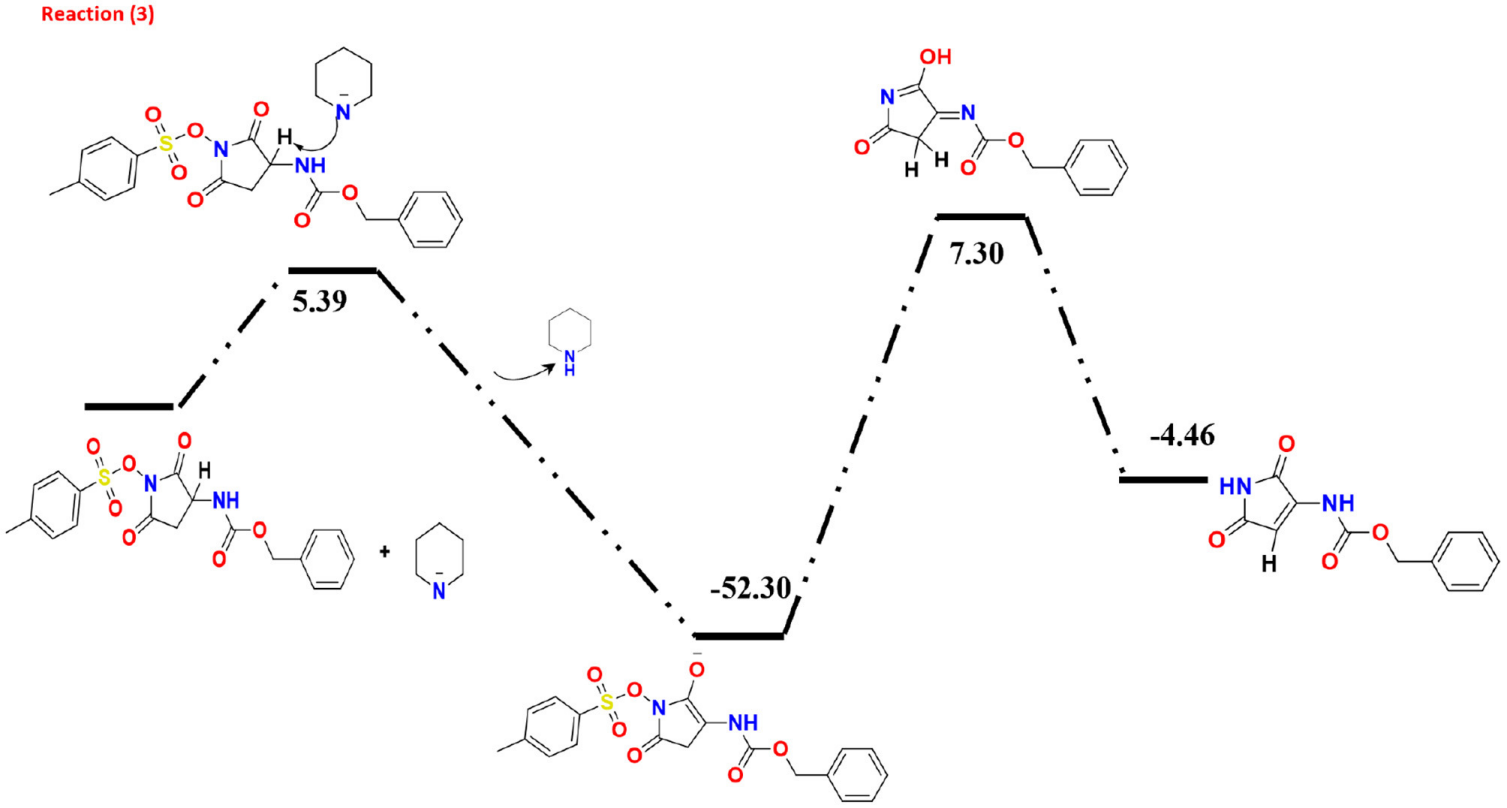
The DFT study of the reaction mechanism of the minor product.

The collected minor product of the secondary amine attack does not show a Lossen-rearrangement. After investigating different models, we suggested the mechanism presented at Figure 5 that started via elimination reaction of hydrogen atom in the pyrrolidine-dione ring due to the basicity character of the amine. Specifically, there are three hydrogen atoms in the ring with positive charge range value, H37, H38, and H47. According the Mulliken and the natural atomic charge (NBO), the H13 has the highest positive value charge could deprotonated easer in the basic media in the presence of the piperidine amine as initial step with energy barrier of 5.39 Kcal/mol. The formed negative charge moved into the intermediate molecules to form a stable product with ΔG = −52.30 Kcal/mol. The next step is the removal of the methylbenzenesulfonate by −45 Kcal/ mol yielding the neutral enol molecule. The final product with keto form was more stable than the enol system by 2.84 Kcal/mol.

### Molecular Docking

The sulfonic esters of N-oxyimides are known for their inhibitory properties against the serine proteases, mainly human leukocyte elastase (HLE) (Neumann and Gütschow, 1994; Abell and Oldham, 1999). This enzyme is an object of clinical interest due to its involvement in pulmonary emphysema and other inflammatory ailments (Hunninghake and Gadek, 1981–1982; Stefanowicz et al., 2006; Tirouvanziam, 2006). There are also reports on the inhibition of other serine proteases, like chymotrypsin and cathepsin G (Wakefield and Wright, 1980).

Our hypothesis is the results of the rearrangement could bind to active site of HLE and have the ability to inhibit of serine proteinases. Molecular docking is common and practical method to study the mechanistic pathway by placing the investigated molecule in the possible binding site of the target specific region (Sharfalddin et al., 2020). Therefore, we test the binding ability of the sulfonic molecules and the product complexes toward the Human Leukocyte Elastase (HLE) by the molecular docking approach.

**Figure 6:**
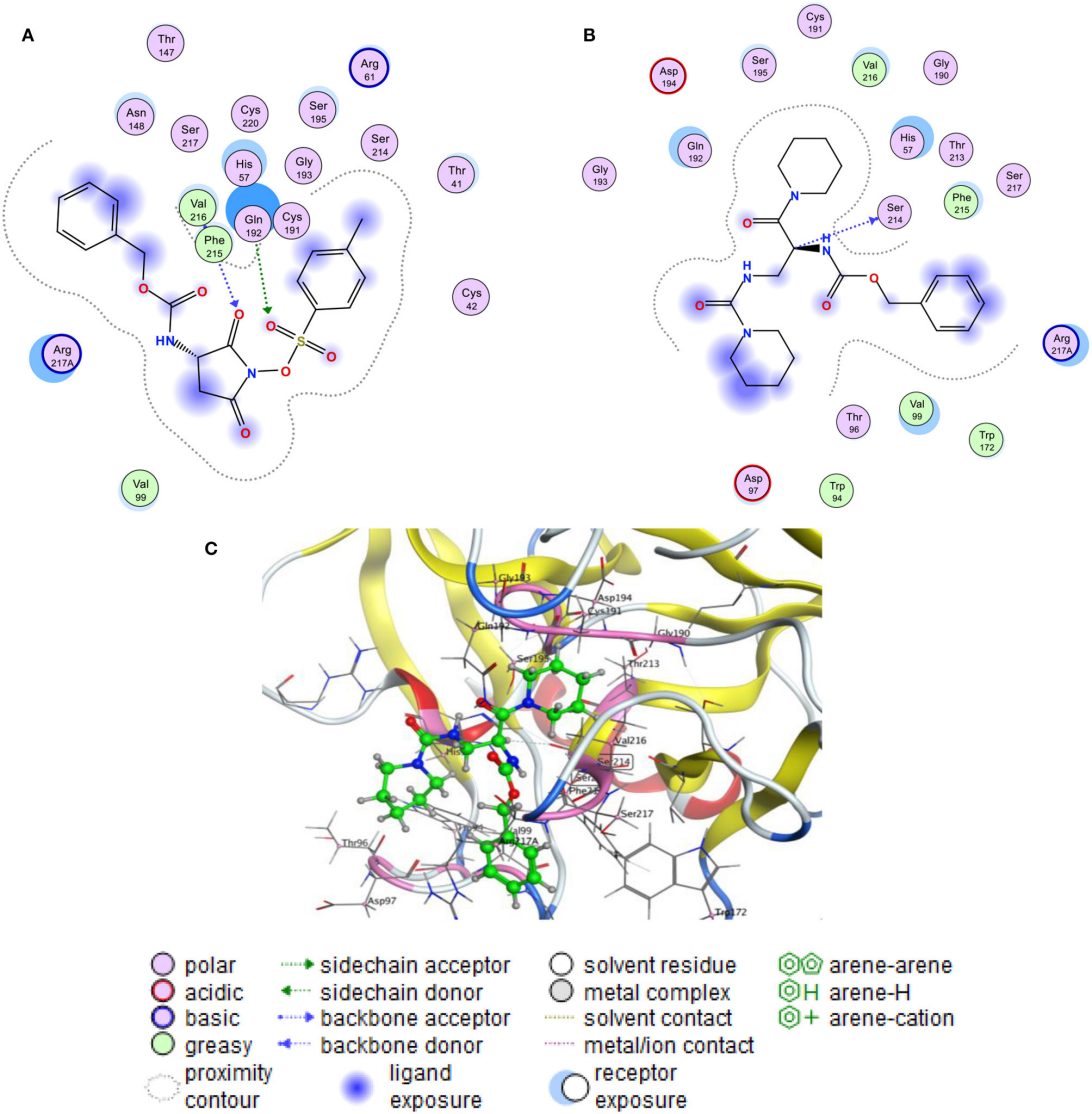
Molecular docking mode and interaction (2D) between the Human Leukocyte Elastase (HLE) and **(A)** sulfonic ester, **(B)** the R1 product and **(C)** the 3D interaction of the R1 product.

To gain insight into the active site, the binding pocket of the co-ligand of HLE has used to generate the active site in the enzyme and to start the docking process. The docking scores of the free binding energy (S) and the root-mean-square deviation of atomic position (RMSD) are present in Table 2. Generally, the highest negative value indicates to stronger binding affinity between the receptor and the tested compounds. The obtained results have been shown the four compounds have reasonable binding with a negative free binding energy value. Meanwhile, the R1 compound presented stronger binding affinity with free binding energy −6.962 kcal/mol while the inhibition of the sulfonic molecule came at the second among the other compounds with −6.548 kcal/mol. Figure 6 showed more details of the interaction that sulfonic esters of N-oxyimides can accept two hydrogens from Gin 192 and Phe 215 nucleoside and might function as inhibitor of the proteinases by assisting the nucleophilic ring opening. interestingly, the resulted compound R1 formed a hydrogen bond by donating the C20 hydrogen with Ser 214 that may could play as another inhibitor. Table 3 shows details of the interaction of the compounds an the HILE enzyme.

**Table 2:** Docking score of the sulfonic molecules and its rection products.

**mol**	**S**	**rmsd_refine**	**E_conf**	**E_place**	**E_score1**	**E_refine**	**E_score2**
R3	−5.82	1.12	−75.12	−65.21	−9.38	−25.97	−5.82
R2	−6.54	1.68	−84.43	−67.22	−9.79	−32.63	−6.54
R1	−6.96	1.92	−67.36	−80.88	−9.12	−30.03	−6.96
sulfonic	−6.55	2.59	2.97	−50.87	−9.43	−35.19	−6.55

**Table 3:** Interactions of complexes sulfonic esters and R1 with the docked HLE enzyme.

	**Ligand**	**Receptor**	**Interaction**	**Distance**	**E (kcal/mol)**
Sulfonic esters	O 28	N VAL 216 (A)	H-acceptor	3.19	−1.1
	O 31	NE2 GLN 192 (A)	H-acceptor	2.97	−2.9
R1	C 20	O SER 214 (A)	H-donor	3.39	−0.5

## Conclusion

Arylosulphonates of N-hydroxyimides undergo diverse reactions with nucleophilic reagents. Herein we confirmed the known reactivity of these compounds, which results in formation of beta-aminoacids. In the case of investigated derivatives of aspartic acid, this reaction yields various derivatives of the non-protein amino acid albizzine (Anderson et al., 1964) as well as 3-amino-3- [(aminocarbonyl)amino] propanoic acid derivatives. Moreover, we discovered a new reaction. In review of N-hydroxyimides arylosulphonates in which elimination of arylosulphonic acid results in formation of the N-protected 3-amino-1H-pyrrole-2,5-dione – the derivative of N-protected dehydroaspartic acid. The hypothetical mechanism of this reaction includes a deprotonation of substrate with amine molecule followed by p-toluenesulphonate elimination and rearrangement of the formed molecule. Details of this process were elucidated by DFT calculations. The calculated results revealed that both reactions could be obtained under the same conditions with small prediction ratio of the R2 when the nucleophilic attack is with C12 due to the positive charge distribution around this atom being higher than C15. Additionally, the steric hindrance resulting from the two hydrogens of the C15 might be another explanation. We have shown previously the suggested bath of the unexpected product of this reaction depending on the same theory. The inhibitory properties of sulfonic esters of N-oxyimide compounds are well-known against human leukocyte elastase. To test this for these products, we applied molecular docking to estimate their binding free energies. Interestingly, one of the products showed practical binding to the human leukocyte elastase (HLE). Thus, further investigation is needed using *in vivo* and *in vitro* to determine these theoretical results.

### Experimental Part

All details concerning the protocol of synthesis, used reagents and obtained data (MS, NMR, X-ray, DFT calculation, Molecular docking calculation) are placed in Supplementary information.

### Computation Method

The DFT calculations have been performed using the Gaussian 09 suite program that employed on the Aziz supercomputing facility in the High Performance Computing Center of King Abdulaziz University (http://hpc.kau.edu.sa). The Becke's three parameter exchange functional (Abell and Oldham, 1999) and the Lee-Yang-Parr correlation functional (Sharfalddin et al., 2020) (Sharfalddin and Hussien, 2021) (B3LYP) method with the 6 311G+(d,p) basis set were used to calculate the geometry optimizations, transition state and the corresponding harmonic vibration frequency. The transition states were characterized by a single imaginary frequency. Solvent effects were included with the CPCM single-point energy calculations on the gas-phase. The Gibbs free energies (ΔG_sol_) in this discussion have obtained in DMSO at 298 K and 1 atm using the value of thermal corrections (G_corr−gas_) in the gas phase. All the optimized geometry files are provided as XYZ files in Supporting Information.

### Molecular Docking Procedure

Molecular operation environment software (MOE) has utilized to dock the complexes toward Human Leukocyte Elastase (PDB = 1EAT). We used the docking protocol that has been descried in our previous work (Sharfalddin et al., 2020). After the crystal structure has downloaded from the PDB www.rcsb.org, the water molecules, co-ligand and the metal ions have been removed. The final structure was obtained after 3D protonation and the correction process. The active binding sites were generated by the MOE site finder to create the dummy sites as binding pocket. The default docking parameters were as follows: triangle matcher for replacing the molecule and London dG for rescoring the docking scores. The DFT optimized structures of the sulfonic reactance and the products molecules have used to generate the best five binding poses with flexible molecules rotation. The hydrogen bonds formation between elastase and investigated compound were used to rank the binding affinity and presented as the free binding energy (S, kcal/mol). The higher negative values of the docking scores were presented along with 2D and 3D structures.

## Data Availability Statement

The original contributions presented in the study are included in the article/Supplementary Material, further inquiries can be directed to the corresponding authors.

## Author Contributions

MK and AAS: performed the experiments, experimental and computational characterization of compounds, and contributed to writing substantially. ŁJ, MC, BS, PS, MJ, and A-HE: performed the part of experimental work and characterization of some compounds. MS: crystallographic data curation and deposition. AAS and MH: performed and designed computational characterization of the compounds and reactions. All authors contributed to paper writing and results and data interpretation at different stages of the manuscript preparation.

## Conflict of Interest

The authors declare that the research was conducted in the absence of any commercial or financial relationships that could be construed as a potential conflict of interest.
