# Supplementary material for: Lossen Rearrangement of p-Toluenesulfonates of N-Oxyimides in Basic Condition, Theoretical Study, and Molecular Docking
Source: Front Chem. 2021 Apr 15;9:662533. doi: 10.3389/fchem.2021.662533 (PMC8082858; doi:10.3389/fchem.2021.662533)
Supplement: Supplementary file 1 [file Data_Sheet_1.docx]

**Supporting Informationa**

**Formation of maleimides by elimination of sulphonic acid molecules from N-oxyimides arylosulphonates**

Monika Kijewska ^2^, Abeer Sharfalddin^3^, Łukasz Jaremko^1^, Marta Cal^2^, Miłosz Siczek^2^, Bartosz Setner^2^, Piotr Stefanowicz^2*^, Mostafa Hussien^3,4^, Abdul-Hamid Emwas^5^, Mariusz Jaremko^1*.^

^1^Division of Biological and Environmental Sciences and Engineering, King Abdullah University of Science and Technology, Saudi Arabia,

^2^Faculty of Chemistry, University of Wrocław, Poland,

^3^Department of Chemistry, Faculty of Sciences, King Abdulaziz University, Saudi Arabia,

^4^Department of Chemistry Faculty of Science, Port Said University, Egypt,

^5^Core Labs, King Abdullah University of Science and Technology (KAUST), Saudi Arabia

* Corresponding authors:

[Mariusz.jaremko@kaust.edu.sa](mailto:Mariusz.jaremko@kaust.edu.sa) and [piotr.stefanowicz@chem.uni.wroc.pl](mailto:piotr.stefanowicz@chem.uni.wroc.pl).

Table of Contents

[**1. General information** 2](#_Toc65144632)

[**2. Material and methods** 2](#_Toc65144633)

[**3. ESI-MS spectra** 12](#_Toc65144634)

[**4. NMR analysis of N-protected 3-amino-1H-pyrrole-2,5-dione (1C1L) (2,5-dioxo-2,5-dihydro-1*H*-pyrrol-3-yl)carbamate** 22](#_Toc65144635)

[**5. Crystal Data** 25](#_Toc65144636)

[**6. Computational calculation data** 36](#_Toc65144637)

# **1. General information**

**Mass spectrometric measurements**

The samples were analyzed using micrOTOF-Q mass spectrometer (Bruker Daltonic, Germany). The micrOTOF-Q instrument equipped with an ESI source with ion funnel, was operated in positive or negative ion mode and calibrated before each analysis with the Tunemix™ mixture (Bruker Daltonics, Germany) in a quadratic method. In MS/MS experiments the collision energy (5-40 eV) was optimized for the best fragmentation. Argon was used as a collision gas. Spectra (positive ion mode) were recorded using methanol with sodium ions at the sample concentration of 0.5 μM. For MS spectra analysis, a Bruker Compass DataAnalysis 4.0 software was used. A Sophisticated Numerical Annotation Procedure (SNAP) algorithm was used for finding peaks. All obtained signals had mass accuracy error in the range of 5 ppm.

**NMR measurements**

NMR is a powerful analytical tool offers a wide range of different experiments that can be used for molecule identification (Aburabie et. al, 2019; Adamu et.al. 2016; Alahmari et. Al. 2019), quantifications (Ali, et. al., 2018; Ali, et. Al, 2020; Aljuhani, et. al., 2019; de Graaf, et. Al., 2003) and structural elucidation (Atiqullah, et. al., 2015; Arda, et.al., 2018; Arrabal-Campos, et.al., 2019; Asghar, et. al., 2018). Thus NMR stand as a standard method in organic chemistry to check reaction kinetics (Mattar, et. al., 2004; Davaasuren, et. al., 2017) and for identification of reaction product (Atiqullah, et. al., 2015; Atiqullah, et. al., 2013; Emwas, et. al., 2020). In the current study we employed both 1D and 2D NMR experiments to identify the final product of the studied reaction. The 1D and 2D spectra of intermediate products were recorded on Bruker Avance 500 MHz spectrometer. The experiments were performed at 25°C. The solvents were selected appropriately to each compound (see synthesis of Asp and Glu derivatives). The concentration of each compound was approx. 10 mg/ml.

# **2. Material and methods**

**Reagents**

All solvents and reagents were used as supplied. The Z-Asp-OH and Z-Glu-OH were purchased from Sigma-Aldrich. All solvents and reagents (acetic anhydride, diethyl ether, petroleum ether, dioxane, ethyl acetate, MeOH, Ethyl Acetate, CHCl_3_, THF, DMSO, triethylamine, piperidine, morhpholine, *m*-toluidine, aniline, propylamine) used in synthesis were purchased from POCH, Sigma-Aldrich or J. T. Baker.

**Synthesis of p-toluenesulfonates of N-oxyimides of aspartic and glutamic acid**

1. **Preparation of the aspartic acid derivatives**

**Scheme S1. Synthesis of p-toluenesulfonates of N-oxyimides of aspartic**

**Aspartic acid anhydride – compound 1A**

To the Z-Asp-OH (5g, 0.02 mol) the acetic anhydride was added (3.5 ml, 0.02 mol) in the molar ratio 1:1. The reaction was allowed to proceed for 2h in the 45°C. After 2h and cooling the product **1A** was crystalized from the mixture. The product 1 A was filtered and wash with diethyl ether. Then the crude product was crystalized from the diethyl ether and crystalized after the addition of petroleum ether. Yield: 73%

*Analytical data:*

[M+Na]^+^ = 272.0489 (calc. *m/z* = 272.0529 for C_12_H_11_NNaO_5_)

**^1^H NMR (CDCl_3,_ 500MHz)** δ = 7.38 (m, 5.47H), 5.62 (d, J=4.3Hz, 1H), 5.14 (m), 5.40 (dt, J=9.7, J=7.1 Hz, 1H), 3.27 (dd, *J*=18.5, *J*=9.9Hz, 1H), 3.15 (dd, *J*=18.5, *J*=7.2 Hz, 1H); 5,50%N, 57.11%C, 4.98%H

T_t_ = 105°C (lit. 109-111°C) (Quesne et al., 1952)

**N-hydroksy aspartic anhydride – compound 1B**

The NH_4_OH*HCl (0.91 g, 0.013 mol) was dissolved in small amount of water (1 ml) and then 2N NaOH (3.25 ml) was added. To the mixtures the 8ml of dioxane was added. To the reaction mixture the N-Z-L-aspartic anhydride (compound **1A**) was added in the portion. The temperature during the reaction was 35°C. After 15 minutes the solution was clear, and the temperature was raised to 60°C. the reaction was allowed to proceed for 2h. The solvent was removed under reduced pressure the obtained oil product was heating to 145°C *in vacuo* (from the 100°C the temperature was raising to 145°C during 15 minutes). The product was heated in 145°C for 30 minutes. The product was extracted with ethyl acetate. The solvent was removed under reduced pressure. The obtain oil product was crystalized from the ethyl acetate. Yield: 71%

*Analytical data:*

[M+Na]^+^ = 287.0604 (calc. *m/z* = 287.0638 for C_12_H_12_N_2_NaO_5_)

**^1^H NMR (DMSO, 500 MHz)** δ = 7.94 (d, *J* = 8.1Hz, 1H), 7.35 (m, 6H), 5.04 (d, *J* = 2.3Hz, 2H), 4.42 (td, *J* = 8.8, *J* = 4.9Hz, 1H), 3.33 (broad s), 2.98 (dd, *J* = 17.2 Hz, *J* = 9.1Hz, 1H), 2.53 (dd**,** *J*=4.9Hz, partially covered with solvent signal); 10.05%N, 53.46%C, 4.60%H

T_t_ = 141-143°C

**Tos - Aspartic acid – compound 1C**

Obtained product (compound **2A**, 2.3 g, 0.009 mol) and p-toluenosulfonic chloride (1.74 g, 0.009 mol) were dissolved in 25 ml of THF and then the 1.25 ml of triethylamine (TEA) was added for 15 minutes. The solution was stirred for 24h. Then the solution was evaporated under reduced pressure. The obtained product was recrystallized form the ethyl acetate. The finally product was obtained according to the procedure previously describe in the literature (Stefanowicz et. al., 2005). Yield: 75 %

*Analytical data:*

[M+Na]^+^ = 441.0776 (calc. *m/z*= 441.0727 for C_19_H_18_N_2_NaO_7_S)

**^1^H NMR(500MHz, CDCl_3_)** δ = 2.54 (s, 3H), 2.90 (dd, *J* = 17.8Hz, *J* = 5.0Hz, 1H), 3.15 (dd, *J* = 17.8Hz, *J* = 9.1Hz, 1H), 4.38 (m, 1H), 5.08 (s, 2H), 5.50 (broad s, 0.84H), 7.33 (m, 7H), 7.92 (d, *J*=8Hz, 2H)

1. **Preparation of glutamic acid derivatives (compound 2A, 2B, 2C)**

**Scheme S2. Synthesis of p-toluenesulfonates of N-oxyimides of glutamic acid**

**Z-glutamic acid anhydride – compound 2A**

The preparation of Z-L-Glutamic acid anhydride was according to the procedure previously described in the literature (Wunsch, 1974). The Z-Glu-OH (10 g, 0.35 mol) was dissolved in 100 ml of THF and then the dicyclohexylocarbodiimide (DCC, 5.54 ml) was added. The reaction was stirred and allowed to proceed for 24h. Then the DCU was filtered from the solution, and the solvent was removed under reduced pressure. Attempts at crystallization of the product failed. The product was used for the further reaction without crystallization. The compound was not stable in a gas phase, so the recording of mass spectra was not possible. Due to the problems with purification, the assignment in NMR spectra was difficult. Yield (without crystallization): 85 %

*Analytical data:*

**^1^H NMR (500MHz, CDCl_3_)** δ = 2.11 (m, 1H), 2.20 (m, 1H), 2.46 (m, 2H), 4.42 (m, 1H), 5.02 (s, 2H), 5.45 (d, *J* = 7.4Hz, 0.75H), 7.34 (m, 5H)

**Z-glutamic acid NHOH – compound 2B**

The NH_4_OH*HCl (0.91 g, 0.013 mol) was dissolved in small amount of water and then triethylamine (2 ml) was added. To the mixtures the 8 ml of dioxane was added. To the reaction mixture the N-Z-L-glutamic anhydride (compound **2A**) was added in the portion. The temperature during the reaction was 35°C. After 15 minutes the solution was clear, and the temperature was raised to 60°C. the reaction was allowed to proceed for 2h. The solvent was removed under reduced pressure and obtained oil product was heating to 145°C *in vacuo* (from the 100°C the temperature was raising to 145 during 15 minutes). The product was heated in 145°C for 30 minutes. The product was extracted with ethyl acetate. The solvent was removed under reduced pressure. The obtain oil product was crystalized from the ethyl acetate. Yield: 75 %

*Analytical data:*

[M+Na]^+^ = 301.0877 (calc. *m/z* = 301.0795 for C_13_H_14_N_2_NaO_5_)

**^1^H NMR (500MHz, DMSO-d_4_)** δ= 1.76 (m, 1H), 1.95 (m, 1H), 2.30 (m, 2H), 3.98 (m, 1H), 7.35 (m, 5H), 7.75 (d, *J* = 8Hz, 1H), 7.79 (d, *J* = 8.7 Hz, 1H).

**Tos – Glutamic acid – compound 2C**

Obtained product (**compound 2B,** 2.4 g, 0.009 mol) and p-toluenosulfonic chloride (1.74 g, 0.009 mol) were dissolved in 25ml of THF and then the 1.25 ml of triethylamine (TEA) was added for 15 minutes. The solution was stirred for 24h. Then the solution was evaporated under reduced pressure. The obtained product was recrystallized form the ethyl acetate. Attempts at crystallization of the product failed. The finally product was oil. Yield (oil product): 55 %.

*Analytical data:*

[M+Na]^+^ = 455.0949 (calc. *m/z* = 455.0883 for C_20_H_20_N_2_NaO_7_S)

**^1^H NMR(500MHz, CDCl_3_)** δ= 2.01 (m, 1H), 2.15 (m, 1H), 2.42(m, 2H), 2.40 (s, 3H), 4.38 (m, 0.75H), 5.04 (s, 2H), 5.72 (d, *J* = 7.8Hz, 1H), 7.28 (m, 9H).

**The reaction of Lossen rearrangement (Lossen rearrangement in basic condition)**

**General protocol:**

The compound **1C** or **2C** (0.0015 mol) was dissolved in 20 ml of solvent (THF or DMSO) and then the **amine** (5 eq) was added. The list of used in the reaction amine is collected in Table S1. The reaction was stirred for 12h at room temperature. Then the solution was evaporated under reduced pressure and product was extracted with ethyl acetate (3 x 10 ml). The organic layers was collected, washed with water and dried over anhydrous MgSO_4_. The organic solvent was removed and the products were analyzed by analytical methods.

Table S1. The collected data of Lossen rearrangement reaction performed on aspartic and glutamic acid derivatives.

| **Amine** | **Products** |
| --- | --- |
| **Aspartic acid derivatives**  ****  **1C** | |
| ****  **1L** | ****  **1C1L** |
| ****  **2L** | ****  **1C2L** |
| ****  **3L** | ****  **1C3L** |
| ****  **4L** | ****  **1C4L** |
| **5L** | **1C5L** |
| ****  **6L** | ****  **1C6L** |
| ****  **7L** | ****  **1C7L** |
| **Glutamic acid derivatives**  ****  **2C** | |
| ****  **2L** | ****  **2C2L** |
| ****  **3L** | ****  **2C3L** |
| ****  **4L** | ****  **2C4L** |
| ****  **5L** | ****  **2C5L** |

Table S2. ESI-MS data for obtained products

| **Nr** | **Amine** | **Product** | **Products**  **(molecular formula)** | | **Products** | | |
| --- | --- | --- | --- | --- | --- | --- | --- |
|  |  |  |  |  | **Calc.**  **[M+Na]^+^** | **Found**  **[M+Na]^+^** | |
| **Aspartic acid derivatives**  **1C** | | | | | | | |
| **1.** | **1L** | **1C1L** | C_12_H_10_N_2_O_4_ | | 269.0533 | | 269.0525 |
| **2.** | **2L** | **1C2L** | C_18_H_28_N_4_O_4_ | | 387.2003 | | 387.1962 |
| **3.** | **3L** | **1C3L** | C_22_H_32_N_4_O_4_ | | 439.2316 | | 439.2418 |
| **4.** | **4L** | **1C4L** | C_20_H_28_N_4_O_6_ | | 443.1901 | | 443.2002 |
| **5.** | **5L** | **1C5L** | C_26_H_28_N_4_O_6_ | | 515.1901 | | 515.1928 |
| **6.** | **6L** | **1C6L** | C_26_H_28_N_4_O_4_ | | 483.2003 | | 483.2034 |
| **7.** | **7L** | **1C7L** | C_24_H_24_N_4_O_4_ | | 455.1689 | | 455.1722 |
| **Glutamic acid derivatives**  ** 2C** | | | | | | | |
| **8.** | **1L** | **2C1L** | C_13_H_12_N_2_O_4_ | 283.0689 | | 283.0704 | |
| **9.** | **2L** | **2C2L** | C_19_H_30_N_4_O_4_ | 401.2159 | | 401.2230 | |
| **10.** | **3L** | **2C3L** | C_23_H_34_N_4_O_4_ | 453.2472 | | 453.2501 | |
| **11.** | **4L** | **2C4L** | C_21_H_30_N_4_O_6_ | 457.2057 | | 457.2176 | |
| **12.** | **5L** | **2C5L** | C_27_H_30_N_4_O_6_ | 529.2058 | | 529.2143 | |

# **3. ESI-MS spectra**

**Fig. S1** ESI-MS spectrum of compound 2C2L

**Fig. S2** ESI-MS spectrum of compound 1C3L (bottom panel – simulated isotopic pattern for desired compound)

**Fig. S3** ESI-MS spectrum for compound **1C3L**

**Fig. S4** ESI-MS spectrum of 1C1L (bottom panel simulated isotopic pattern for proposed compound)

**Fig. S5** ESI-MS spectrum of compound 1C3L (bottom panel – simulated isotopic pattern for desired compound)

**Fig. S6** ESI-MS/MS spectrum of compound 1C4L

**Fig. S7** ESI-MS/MS spectrum of compound 1C5L

**Fig. S8** ESI-MS/MS spectrum of compound 2C5L

**Fig. S9** ESI-MS/MS spectrum of compound 1C2L

**Fig. S10** ESI-MS/MS spectrum of compound1C3L

# **4. NMR analysis of N-protected 3-amino-1H-pyrrole-2,5-dione (1C1L) (2,5-dioxo-2,5-dihydro-1*H*-pyrrol-3-yl)carbamate**

Fig. S11 The schematic presentation of compound N-protected 3-amino-1H-pyrrole-2,5-dione (1C1L). The letters A-D are marked the proton.

Analytical data

**[M+Na]^+^** = 269.0586 (calcd. *m/z* = 269.0533 for C_12_H_10_N_2_O_4_Na )

**^1^H NMR (500MHz, DMSO-d_4_)** δ = 5.21 (s, 2H, C), 6.04 (s, 1H, D), 7.34 (m, 1H, B), 7.38 (m, 4H, A)

**^13^C NMR (125MHz, DMSO-d_4_)** δ = 68.0 (C), 103.6 (D), 128.31 (A), 128.83 (B)

Due to the containing of water in the DMSO-d_4_ the protons associated with the nitrogens were exchanged and we do not see them on the spectrum. The N-benzyloxycarbonyl-amino acids derivatives are well known and described in the literature (**Figure S11**, protons **A**, **B and C**) (Cal, et. al., 2013). The assignment for proton from double bond were corrected with this presented for analogical compounds (Silverstein et. al., 2005). The ^1^H NMR is presented in **Figure S12**.

Fig. S12 The ^1^H NMR (500MHz, DMSO-d_4_) measured for compound N-protected 3-amino-1H-pyrrole-2,5-dione (1C1L).


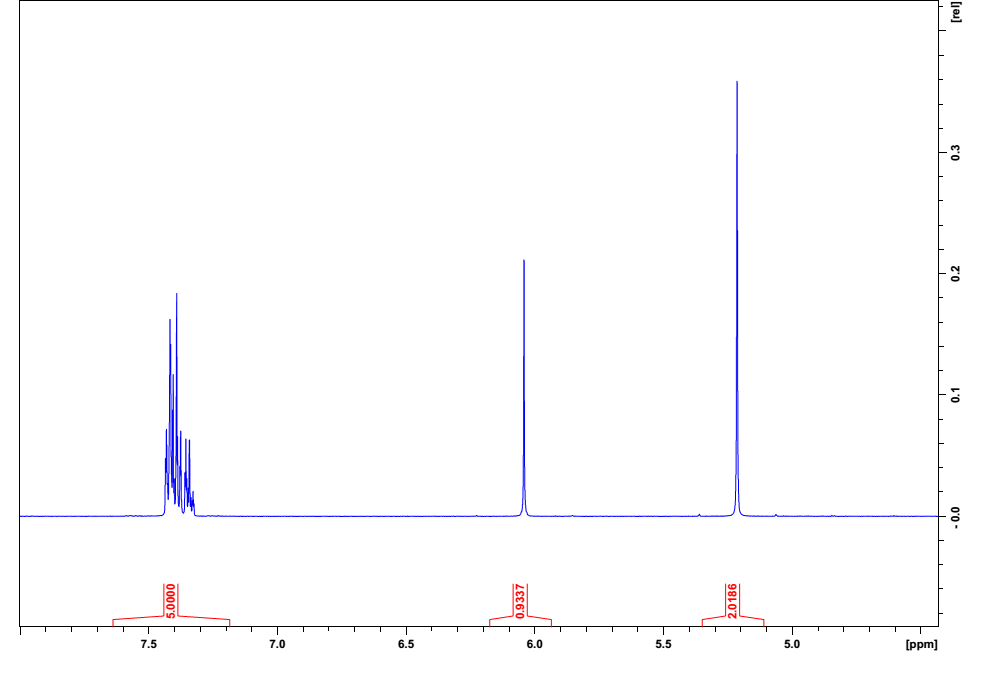


**Fig. S13** 1H NMR spectrum of 1C1L (zoom region 4-8 ppm)


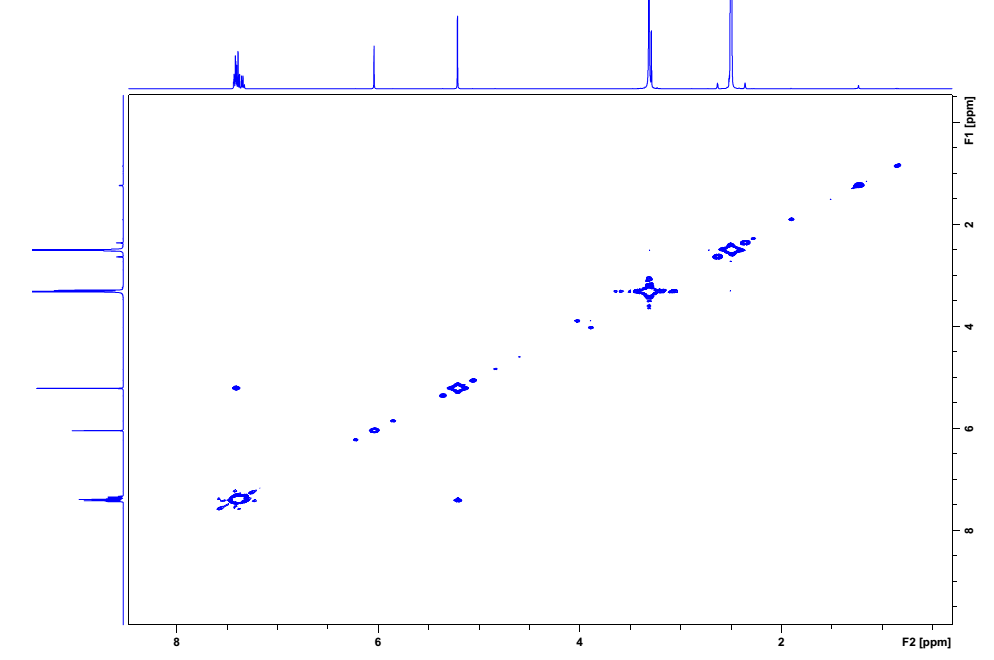


**Fig. S14** 2D COSY spectrum of 1C1L


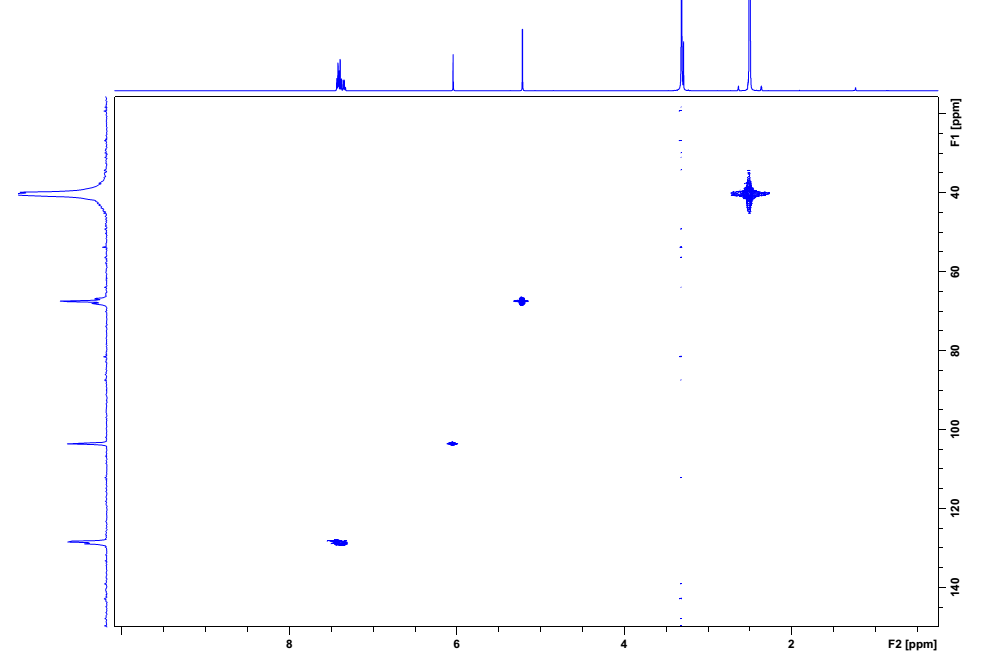


**Fig. S15** HMBC spectrum of 1C1L

# **5. Crystal Data**

*Computing details*

Program(s) used to solve structure: *SHELXS97* (Sheldrick, 1990); program(s) used to refine structure: *SHELXL97* (Sheldrick, 1997).

Diffraction data were collected on a Kuma KM-4 diffractometer equipped with graphite-monochromataized Mo K_α_ radiation (λ = 0.71073 Å) and Sapphire 2 CCD detector for 1C1L. Diffraction data for 1C1L·DMSO were collected on an Xcalibur PX equipped with graphite-monochromataized Cu K_α_ radiation (λ = 1.54184 Å Å) and Onyx CCD detector. Data were processed using the CrystAlisPro software. The structures were solved by direct methods with SHELXS (G.M. Sheldrick, Acta Crystallogr., Sect. A 64 (2007) 112-122) and refined by full-matrix least-squares methods based F^2^ using SHELXL(G.M. Sheldrick, Acta Cryst. Sect. C: Struct. Chem. 71 (2015) 3–8.). The H atoms bound to C atoms were placed in the geometrically idealized positions and treated in riding mode, with C-H = 0.95Å and U_iso_(H) = 1.2U_eq_(C) for C-H groups, and C-H = 0.98Å and U_iso_(H) = 1.5U_eq_(C) for CH_3_ groups, while the N-bound H atoms were refined freely. CCDC- 2064551 (for 1C1L·DMSO), and 2064552 (for 1C1L) contain the supplementary crystallographic data for this paper. **These data can be obtained free of charge from The Cambridge Crystallographic Data Center via www.ccdc.cam.ac.uk/data_request/cif.**

**Table S3. Experimental details**

| Compound reference | 1C1L·DMSO | 1C1L |
| --- | --- | --- |
| Chemical formula | C_12_H_10_N_2_O_4_•C_2_H_6_OS | C_12_H_10_N_2_O_4_ |
| Formula Mass | 324.35 | 246.22 |
| Crystal system | monoclinic | monoclinic |
| *a*/Å | 10.888(3) | 15.860(5) |
| *b*/Å | 11.090(3) | 8.000(4) |
| *c*/Å | 13.002(4) | 35.548(9) |
| *β*/° | 92.16(3) | 97.10(3) |
| Unit cell volume/Å ^3^ | 1568.8(8) | 4476(3) |
| Temperature/K | 100(2) | 100(2) |
| Space group | *P*2_1_*/c* | *C*2*/c* |
| No. of formula units per unit cell, *Z* | 4 | 16 |
| Radiation type | CuKα | MoKα |
| No. of reflections measured | 9101 | 10950 |
| No. of independent reflections | 3169 | 5044 |
| *R_int_* | 0.0368 | 0.0357 |
| Final *R_1_* values (*I* > 2*σ*(*I*)) | 0.0598 | 0.0506 |
| Final *wR*(*F*^2^) values (*I* > 2*σ*(*I*)) | 0.1709 | 0.1218 |
| Final *R_1_* values (all data) | 0.0646 | 0.0670 |
| Final *wR*(*F*^2^) values (all data) | 0.1845 | 0.1330 |

**Table S4. Hydrogen-bond geometry (Å, º) 1C1L·DMSO**

| *D*—H···*A* | *D*—H | H···*A* | *D*···*A* | *D*—H···*A* |
| --- | --- | --- | --- | --- |
| N1—H1···O1*S* | 0.88 (3) | 1.98 (3) | 2.828 (2) | 163 (3) |
| N2—H2···O1*S*^i^ | 0.88 (3) | 1.92 (3) | 2.791 (3) | 177 (3) |
| C7—H7*B*···O3^ii^ | 0.99 | 2.57 | 3.390 (3) | 141 |
| C1*S*—H1*SB*···O4^iii^ | 0.98 | 2.42 | 3.339 (3) | 156 |

Symmetry codes: (i) −*x*, *y*+1/2, −*z*+3/2; (ii) −*x*, −*y*, −*z*+2; (iii) *x*+1, *y*, *z*.

**Table S5. Hydrogen-bond geometry (Å, º) 1C1L**

| *D*—H···*A* | *D*—H | H···*A* | *D*···*A* | *D*—H···*A* |
| --- | --- | --- | --- | --- |
| N1*A*—H1*A*···O2*B* | 0.92 (2) | 1.95 (2) | 2.854 (2) | 169 (2) |
| N2*A*—H2*A*···O3*B*^i^ | 0.88 (2) | 1.99 (2) | 2.864 (2) | 169 (2) |
| N1*B*—H1*B*···O3*A* | 0.93 (2) | 2.04 (2) | 2.955 (2) | 167.3 (19) |
| N2*B*—H2*B*···O4*A*^i^ | 0.91 (2) | 1.94 (2) | 2.838 (2) | 171 (2) |

Symmetry code: (i) −*x*, −*y*+2, −*z*.

**Table S6. Selected geometric parameters(Å, º) for 1C1L**

| O1A-C8A | 1.337(2) | O1B-C8B | 1.328(2) |
| --- | --- | --- | --- |
| O1A-C7A | 1.4537(19) | O1B-C7B | 1.467(2) |
| O2A-C8A | 1.201(2) | O2B-C8B | 1.211(2) |
| O3A-C10A | 1.215(2) | O3B-C10B | 1.212(2) |
| O4A-C11A | 1.222(2) | O4B-C11B | 1.212(2) |
| N1A-C9A | 1.363(2) | N1B-C9B | 1.373(2) |
| N1A-C8A | 1.385(2) | N1B-C8B | 1.375(2) |
| N1A-H1A | 0.92(2) | N1B-H1B | 0.93(2) |
| N2A-C10A | 1.367(2) | N2B-C10B | 1.365(2) |
| N2A-C11A | 1.394(2) | N2B-C11B | 1.401(2) |
| N2A-H2A | 0.88(2) | N2B-H2B | 0.91(2) |
| C1A-C2A | 1.392(2) | C1B-C6B | 1.385(3) |
| C1A-C6A | 1.396(2) | C1B-C2B | 1.386(3) |
| C1A-C7A | 1.497(2) | C1B-C7B | 1.498(2) |
| C2A-C3A | 1.388(3) | C2B-C3B | 1.391(3) |
| C3A-C4A | 1.383(3) | C3B-C4B | 1.375(4) |
| C4A-C5A | 1.386(3) | C4B-C5B | 1.384(4) |
| C5A-C6A | 1.383(3) | C5B-C6B | 1.385(3) |
| C9A-C12A | 1.345(2) | C9B-C12B | 1.340(2) |
| C9A-C10A | 1.512(2) | C9B-C10B | 1.509(2) |
| C11A-C12A | 1.472(2) | C11B-C12B | 1.478(2) |
| C8A-O1A-C7A | 113.84(12) | C8B-O1B-C7B | 117.20(13) |
| C9A-N1A-C8A | 120.71(14) | C9B-N1B-C8B | 127.13(15) |
| C10A-N2A-C11A | 109.94(14) | C10B-N2B-C11B | 110.46(14) |
| C2A-C1A-C6A | 119.00(16) | C6B-C1B-C2B | 119.72(19) |
| C2A-C1A-C7A | 123.16(15) | C6B-C1B-C7B | 120.73(17) |
| C6A-C1A-C7A | 117.83(15) | C2B-C1B-C7B | 119.5(2) |
| C3A-C2A-C1A | 120.01(17) | C1B-C2B-C3B | 119.6(3) |
| C4A-C3A-C2A | 120.50(18) | C4B-C3B-C2B | 120.3(2) |
| C3A-C4A-C5A | 119.92(18) | C3B-C4B-C5B | 120.4(2) |
| C6A-C5A-C4A | 119.77(17) | C4B-C5B-C6B | 119.4(3) |
| C5A-C6A-C1A | 120.80(16) | C5B-C6B-C1B | 120.5(2) |
| O1A-C7A-C1A | 109.00(13) | O1B-C7B-C1B | 108.95(14) |
| O2A-C8A-O1A | 125.96(15) | O2B-C8B-O1B | 126.02(16) |
| O2A-C8A-N1A | 123.99(16) | O2B-C8B-N1B | 122.43(16) |
| O1A-C8A-N1A | 110.04(14) | O1B-C8B-N1B | 111.54(14) |
| C12A-C9A-N1A | 133.20(15) | C12B-C9B-N1B | 135.53(16) |
| C12A-C9A-C10A | 108.54(15) | C12B-C9B-C10B | 108.73(14) |
| N1A-C9A-C10A | 118.25(14) | N1B-C9B-C10B | 115.74(15) |
| O3A-C10A-N2A | 127.28(15) | O3B-C10B-N2B | 128.07(16) |
| O3A-C10A-C9A | 126.55(16) | O3B-C10B-C9B | 125.91(15) |
| N2A-C10A-C9A | 106.16(13) | N2B-C10B-C9B | 106.02(14) |
| O4A-C11A-N2A | 124.37(15) | O4B-C11B-N2B | 124.05(16) |
| O4A-C11A-C12A | 127.51(15) | O4B-C11B-C12B | 128.83(17) |
| N2A-C11A-C12A | 108.12(14) | N2B-C11B-C12B | 107.13(14) |
| C9A-C12A-C11A | 107.22(14) | C9B-C12B-C11B | 107.65(15) |
| C6A-C1A-C2A-C3A | 0.7(3) | C6B-C1B-C2B-C3B | −0.1(3) |
| C7A-C1A-C2A-C3A | 179.3(2) | C7B-C1B-C2B-C3B | 178.5(2) |
| C1A-C2A-C3A-C4A | 0.1(4) | C1B-C2B-C3B-C4B | −0.6(4) |
| C2A-C3A-C4A-C5A | −0.8(4) | C2B-C3B-C4B-C5B | 1.0(4) |
| C3A-C4A-C5A-C6A | 0.8(4) | C3B-C4B-C5B-C6B | −0.7(4) |
| C4A-C5A-C6A-C1A | −0.1(3) | C4B-C5B-C6B-C1B | 0.0(3) |
| C2A-C1A-C6A-C5A | −0.6(3) | C2B-C1B-C6B-C5B | 0.4(3) |
| C7A-C1A-C6A-C5A | −179.34(19) | C7B-C1B-C6B-C5B | −178.24(17) |
| C8A-O1A-C7A-C1A | −162.85(15) | C8B-O1B-C7B-C1B | −109.47(17) |
| C2A-C1A-C7A-O1A | 7.4(3) | C6B-C1B-C7B-O1B | 83.4(2) |
| C6A-C1A-C7A-O1A | −173.98(16) | C2B-C1B-C7B-O1B | −95.2(2) |
| C7A-O1A-C8A-O2A | 1.2(2) | C7B-O1B-C8B-O2B | −1.1(3) |
| C7A-O1A-C8A-N1A | −179.56(14) | C7B-O1B-C8B-N1B | 179.97(14) |
| C9A-N1A-C8A-O2A | −3.5(3) | C9B-N1B-C8B-O2B | 171.26(17) |
| C9A-N1A-C8A-O1A | 177.22(15) | C9B-N1B-C8B-O1B | −9.8(3) |
| C8A-N1A-C9A-C12A | −0.2(3) | C8B-N1B-C9B-C12B | 4.2(3) |
| C8A-N1A-C9A-C10A | −179.14(15) | C8B-N1B-C9B-C10B | −176.06(16) |
| C11A-N2A-C10A-O3A | −179.67(17) | C11B-N2B-C10B-O3B | −178.94(18) |
| C11A-N2A-C10A-C9A | 0.80(19) | C11B-N2B-C10B-C9B | 0.61(19) |
| C12A-C9A-C10A-O3A | 179.27(18) | C12B-C9B-C10B-O3B | 179.47(18) |
| N1A-C9A-C10A-O3A | −1.5(3) | N1B-C9B-C10B-O3B | −0.4(3) |
| C12A-C9A-C10A-N2A | −1.2(2) | C12B-C9B-C10B-N2B | −0.1(2) |
| N1A-C9A-C10A-N2A | 177.99(15) | N1B-C9B-C10B-N2B | −179.93(15) |
| C10A-N2A-C11A-O4A | 179.26(17) | C10B-N2B-C11B-O4B | 179.16(18) |
| C10A-N2A-C11A-C12A | −0.17(19) | C10B-N2B-C11B-C12B | −0.9(2) |
| N1A-C9A-C12A-C11A | −177.95(18) | N1B-C9B-C12B-C11B | 179.4(2) |
| C10A-C9A-C12A-C11A | 1.1(2) | C10B-C9B-C12B-C11B | −0.4(2) |
| O4A-C11A-C12A-C9A | 179.98(18) | O4B-C11B-C12B-C9B | −179.2(2) |
| N2A-C11A-C12A-C9A | −0.6(2) | N2B-C11B-C12B-C9B | 0.8(2) |

**Table S7. Selected geometric parameters(Å, º) for 1C1L·DMSO**

| O1-C8 | 1.344(3) | C1-C2 | 1.395(3) |
| --- | --- | --- | --- |
| O1-C7 | 1.445(2) | C1-C7 | 1.510(3) |
| O2-C8 | 1.203(3) | C2-C3 | 1.386(3) |
| O3-C10 | 1.210(3) | C3-C4 | 1.387(4) |
| O4-C11 | 1.217(3) | C4-C5 | 1.388(3) |
| N1-C9 | 1.371(3) | C5-C6 | 1.390(3) |
| N1-C8 | 1.373(3) | C9-C12 | 1.339(3) |
| N1-H1 | 0.88(3) | C9-C10 | 1.508(3) |
| N2-C10 | 1.366(3) | C11-C12 | 1.477(3) |
| N2-C11 | 1.398(3) | S1S-O1S | 1.5105(16) |
| N2-H2 | 0.88(3) | S1S-C1S | 1.776(3) |
| C1-C6 | 1.391(3) | S1S-C2S | 1.795(3) |
| C8-O1-C7 | 114.92(16) | C3-C2-C1 | 120.0(2) |
| C9-N1-C8 | 123.26(18) | O1-C8-N1 | 108.79(17) |
| C9-N1-H1 | 118.0(19) | C12-C9-N1 | 134.4(2) |
| C8-N1-H1 | 118.0(19) | C12-C9-C10 | 108.88(18) |
| C10-N2-C11 | 110.15(18) | N1-C9-C10 | 116.70(18) |
| C10-N2-H2 | 123.2(19) | O3-C10-N2 | 128.04(19) |
| C11-N2-H2 | 124.7(19) | O3-C10-C9 | 125.88(19) |
| C6-C1-C2 | 119.4(2) | N2-C10-C9 | 106.07(17) |
| C6-C1-C7 | 118.07(19) | O4-C11-N2 | 124.0(2) |
| C2-C1-C7 | 122.56(19) | O4-C11-C12 | 128.5(2) |
|  |  |  |  |
| C2-C3-C4 | 120.7(2) | C5-C6-C1 | 120.3(2) |
| C3-C4-C5 | 119.4(2) | O1-C7-C1 | 108.25(17) |
| C4-C5-C6 | 120.2(2) |  |  |
|  |  |  |  |
| N2-C11-C12 | 107.57(18) | O1S-S1S-C1S | 105.87(12) |
| C9-C12-C11 | 107.31(19) | O1S-S1S-C2S | 104.94(14) |
| C6-C1-C2-C3 | −1.0(3) | C1S-S1S-C2S | 97.91(15) |
| C7-C1-C2-C3 | 178.23(19) | C8-N1-C9-C12 | −2.0(4) |
| C1-C2-C3-C4 | 0.4(3) | C8-N1-C9-C10 | 177.40(18) |
| C2-C3-C4-C5 | 0.4(4) | C11-N2-C10-O3 | −179.4(2) |
| C3-C4-C5-C6 | −0.6(4) | C11-N2-C10-C9 | −0.4(2) |
| C4-C5-C6-C1 | 0.0(3) | C12-C9-C10-O3 | 178.5(2) |
| C2-C1-C6-C5 | 0.8(3) | N1-C9-C10-O3 | −1.0(3) |
| C7-C1-C6-C5 | −178.5(2) | C12-C9-C10-N2 | −0.5(2) |
| C8-O1-C7-C1 | 175.11(17) | N1-C9-C10-N2 | 179.95(17) |
| C6-C1-C7-O1 | −160.50(18) | C10-N2-C11-O4 | −179.1(2) |
| C2-C1-C7-O1 | 20.3(3) | C10-N2-C11-C12 | 1.1(2) |
| C7-O1-C8-O2 | 5.9(3) | N1-C9-C12-C11 | −179.4(2) |
| C7-O1-C8-N1 | −175.24(16) | C10-C9-C12-C11 | 1.1(2) |
| C9-N1-C8-O2 | −3.7(4) | O4-C11-C12-C9 | 178.8(2) |
| C9-N1-C8-O1 | 177.34(18) | N2-C11-C12-C9 | −1.4(2) |
|  |  |  |  |


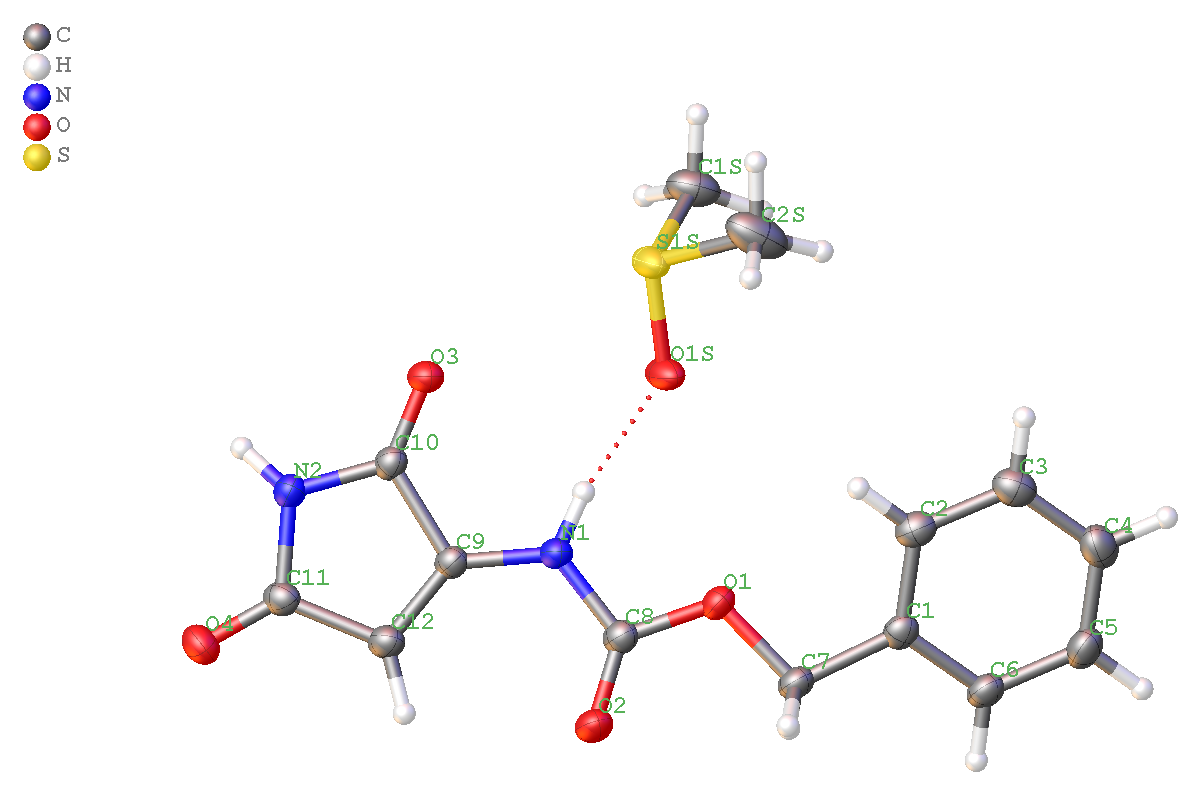


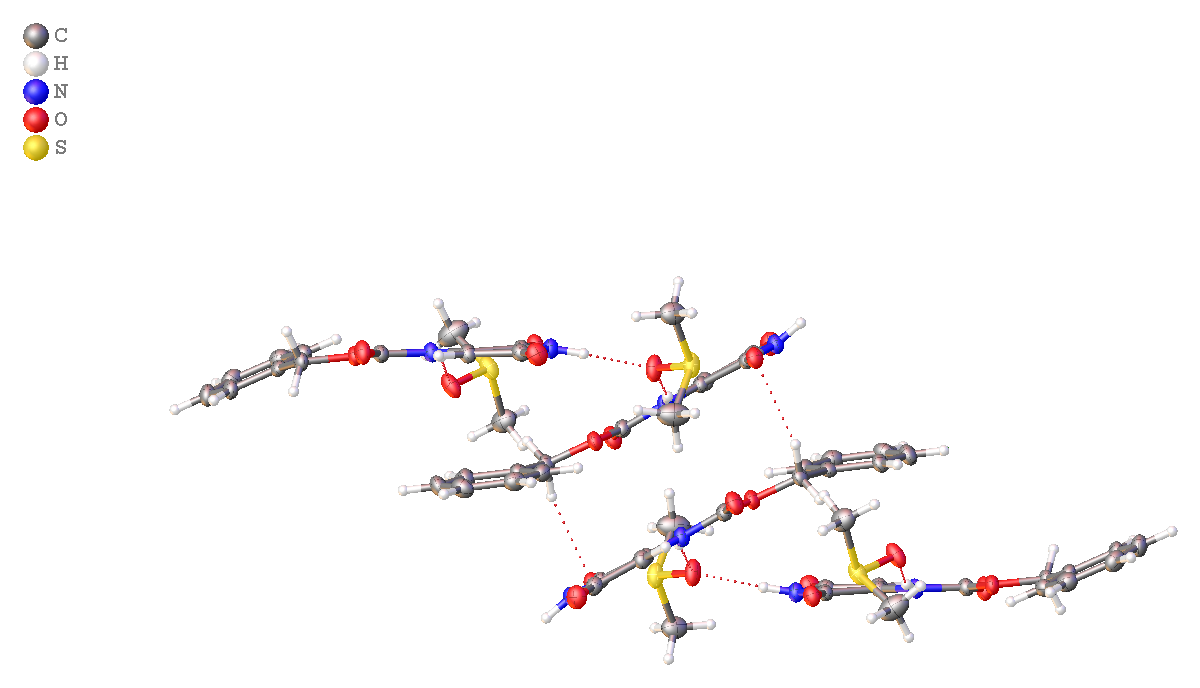


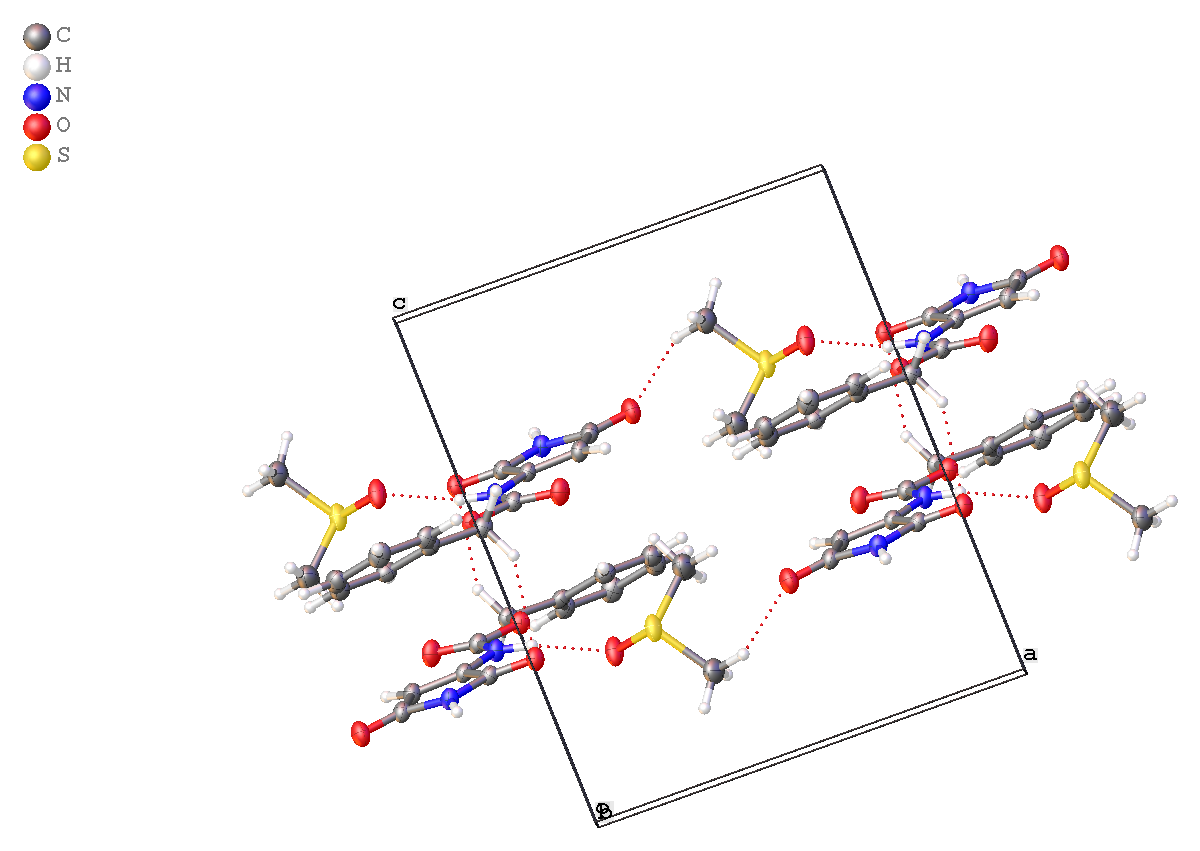


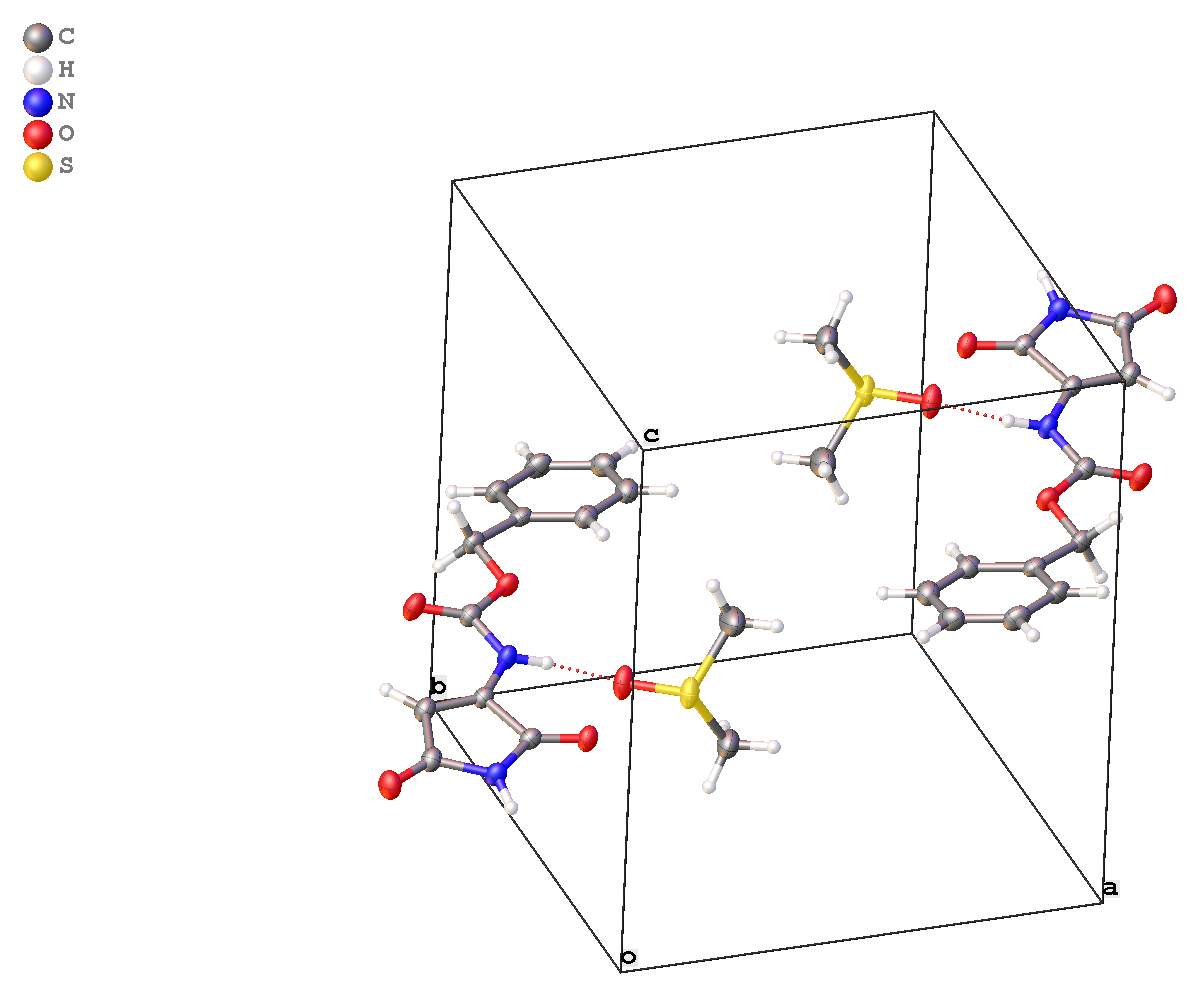


**Fig. S16 The arrangement of molecules (1Cl1L·DMSO) in the crystal structures**


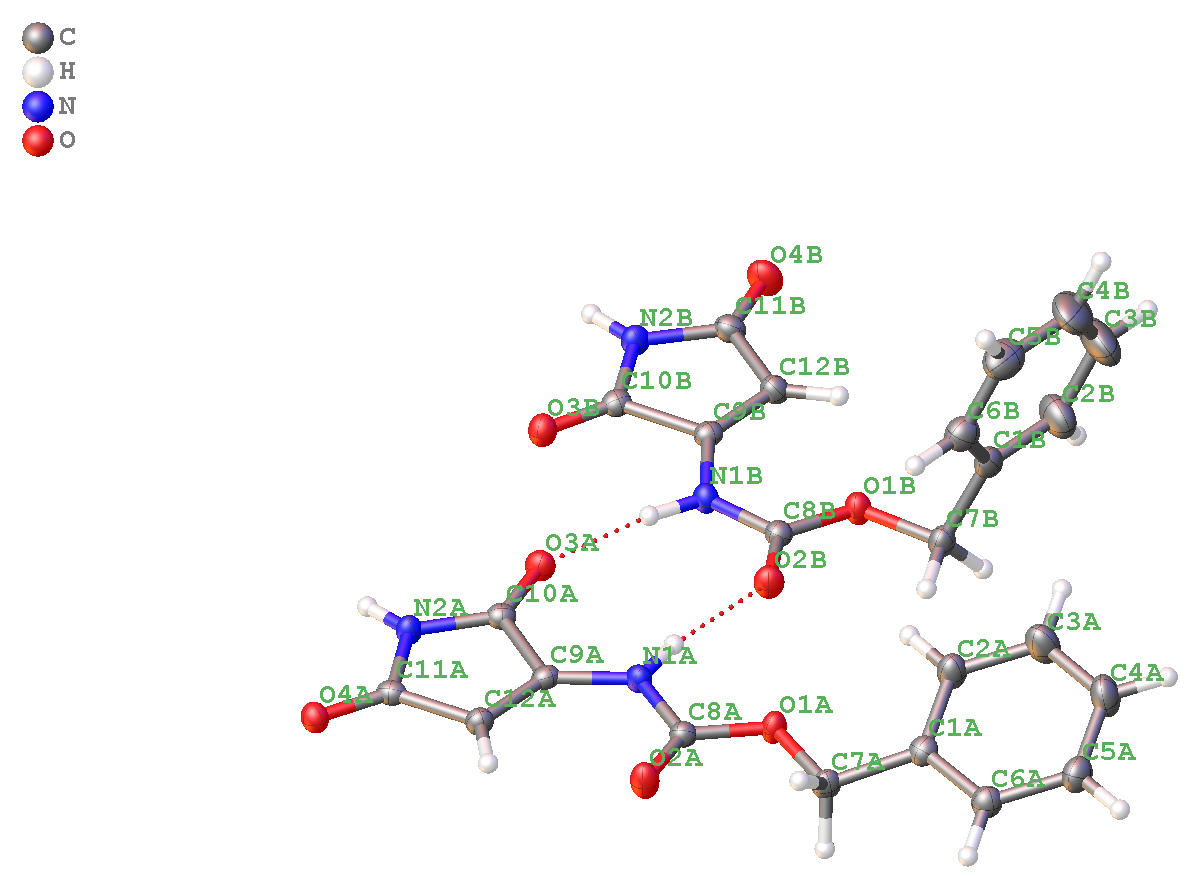


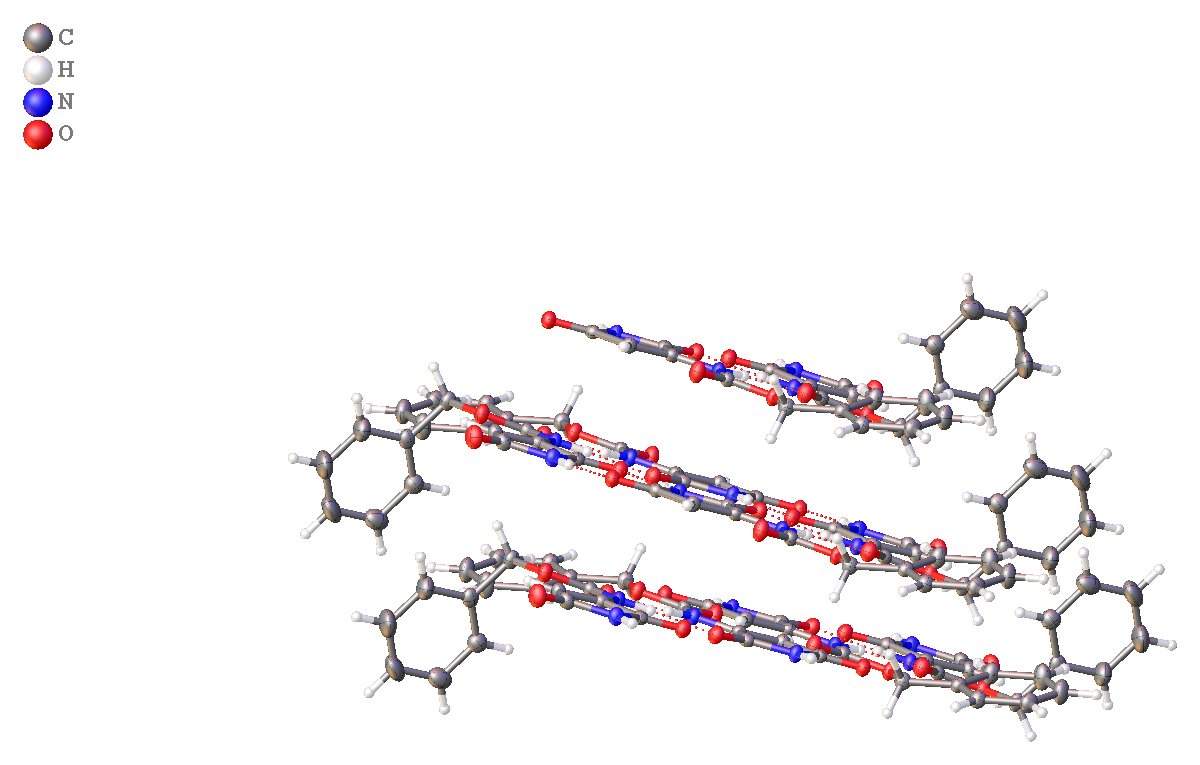


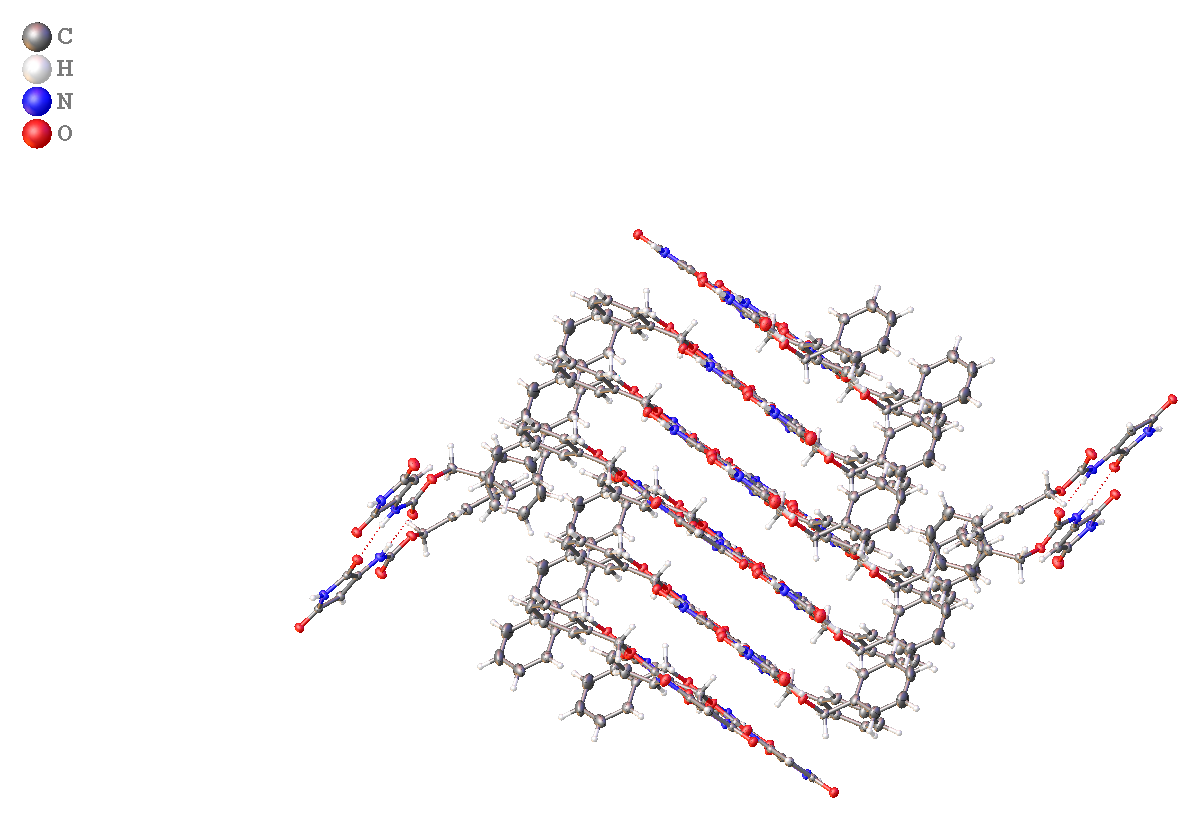


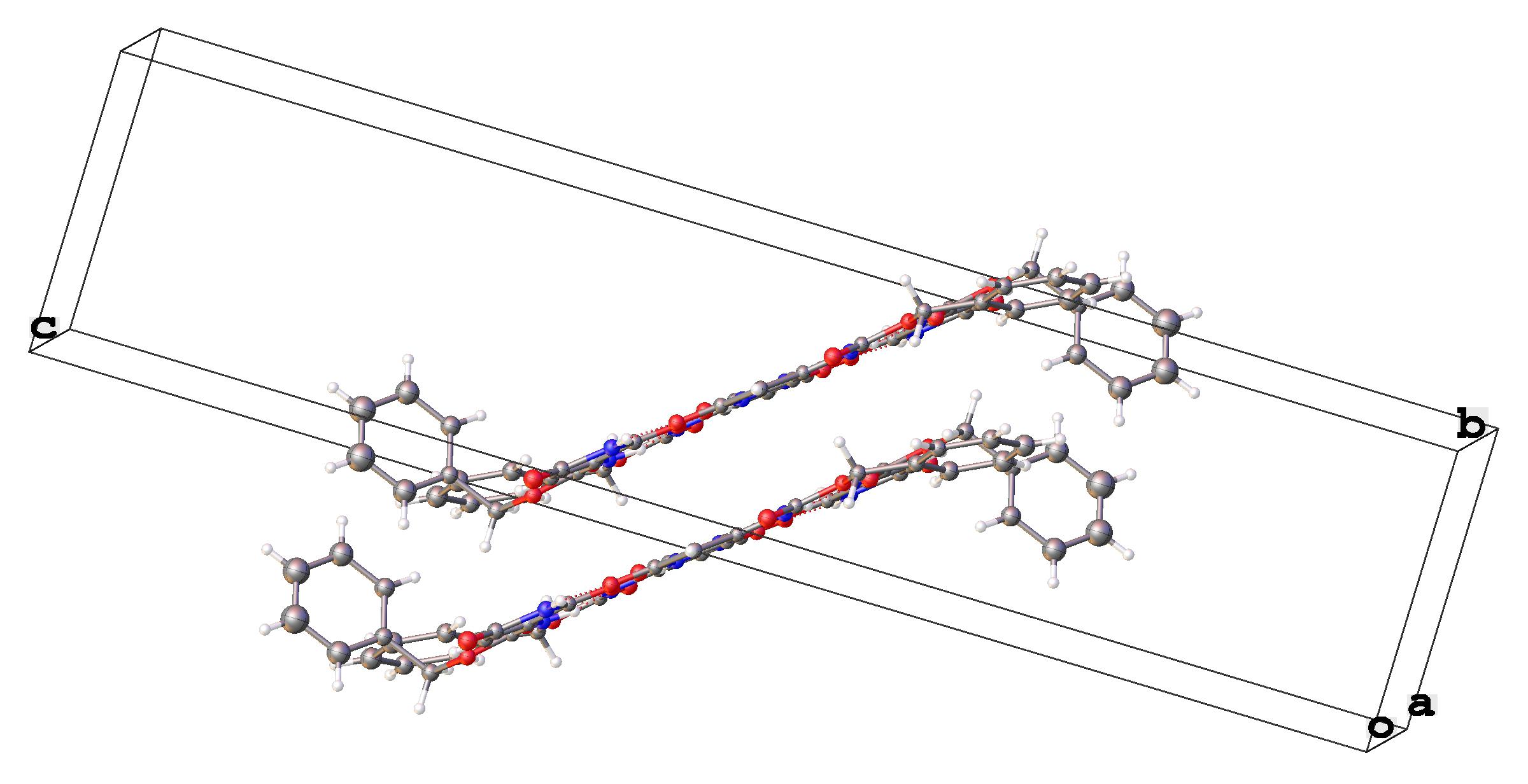


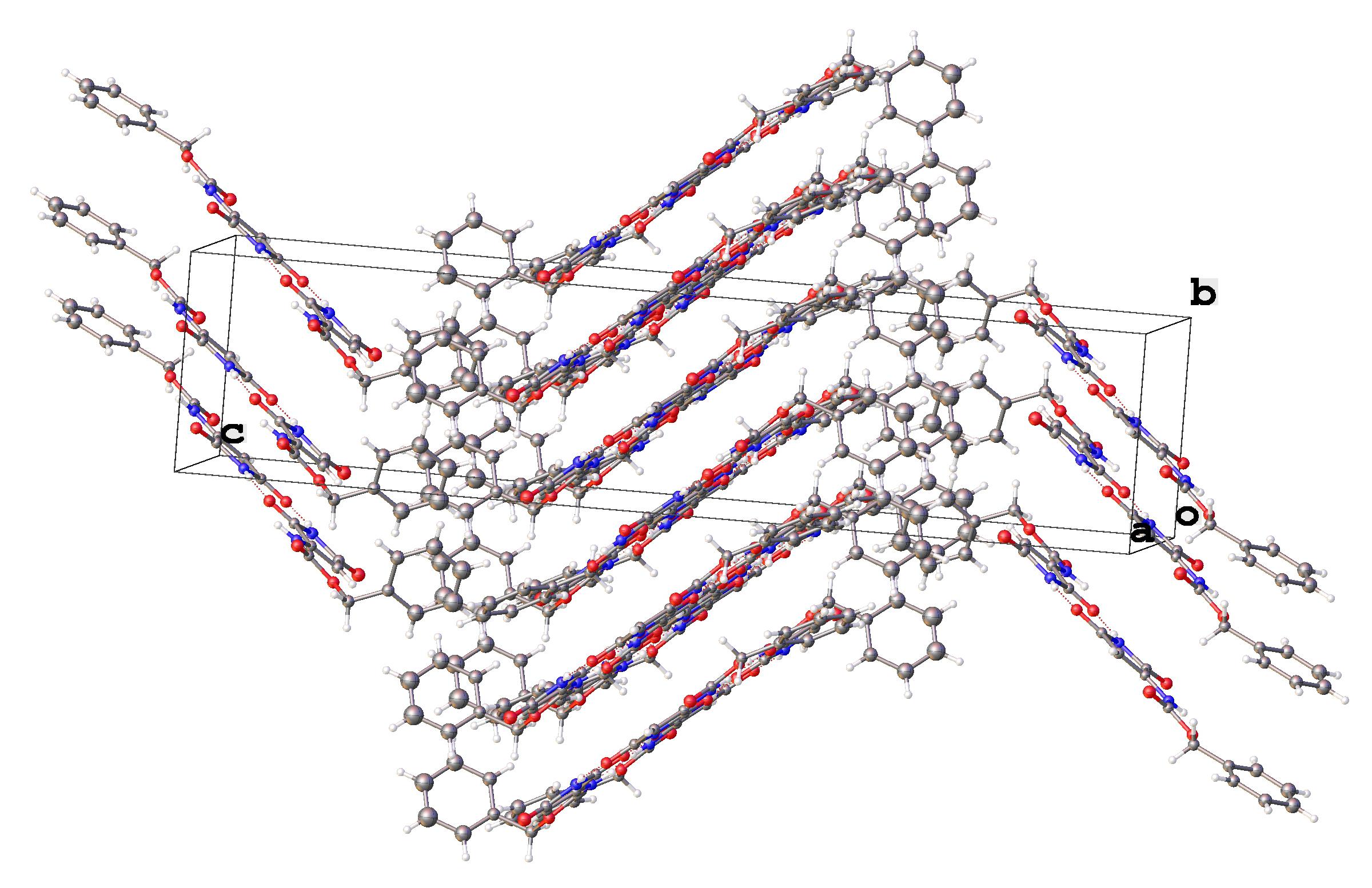


**Fig. S17 The arrangement of molecules (1Cl1L) in the crystal structures**

# **6. Computational calculation data**

**Sulfonic reactant**

-------------------------------------------------------------------

Center Atomic Forces (Hartrees/Bohr)

Number Number X Y Z

-------------------------------------------------------------------

1 6 0.000000176 0.000002051 0.000004793

2 6 -0.000001021 0.000001705 0.000006428

3 6 -0.000002000 0.000002103 0.000005694

4 6 -0.000001340 0.000001180 0.000003642

5 6 0.000000558 0.000001908 0.000002385

6 6 -0.000001923 0.000004468 0.000002583

7 6 0.000000470 0.000001580 0.000002324

8 8 0.000001966 0.000003105 -0.000004280

9 6 0.000002319 0.000004528 0.000003293

10 8 -0.000004110 0.000000445 -0.000001419

11 7 0.000006830 0.000001257 -0.000000651

12 6 -0.000001572 0.000005137 -0.000005166

13 6 0.000001448 -0.000003337 -0.000001292

14 6 0.000005097 0.000001506 -0.000008508

15 6 -0.000001975 -0.000000879 -0.000006120

16 7 0.000000162 0.000000611 -0.000001841

17 8 0.000002735 -0.000003041 -0.000006678

18 8 0.000001168 -0.000000678 -0.000000175

19 8 0.000002353 -0.000002177 -0.000001969

20 16 0.000000911 -0.000002906 -0.000008394

21 8 -0.000000856 -0.000005863 -0.000003795

22 8 0.000001161 -0.000004037 -0.000000443

23 6 0.000000599 -0.000002517 -0.000000838

24 6 -0.000001562 -0.000002911 -0.000003220

25 6 -0.000001168 -0.000003077 0.000001782

26 6 0.000001399 -0.000001207 -0.000000195

27 6 -0.000001461 -0.000002008 -0.000000237

28 6 -0.000000934 -0.000001732 -0.000000006

29 6 -0.000000407 -0.000001074 0.000001150

30 1 -0.000000696 0.000003551 0.000005228

31 1 -0.000002001 0.000002246 0.000007356

32 1 -0.000002067 0.000001206 0.000006606

33 1 -0.000001011 0.000001025 0.000003728

34 1 0.000000158 0.000002004 0.000001407

35 1 0.000001491 0.000004098 0.000000470

36 1 -0.000002580 -0.000000798 -0.000000762

37 1 0.000001407 -0.000000510 -0.000004599

38 1 0.000000673 -0.000002136 -0.000003223

39 1 0.000000004 -0.000003926 -0.000003194

40 1 -0.000000520 -0.000002976 -0.000001449

41 1 -0.000001079 -0.000000511 0.000002460

42 1 -0.000000149 -0.000001919 0.000000466

43 1 -0.000003316 -0.000002046 0.000004054

44 1 -0.000000328 0.000001092 0.000003591

45 1 -0.000000864 -0.000001571 0.000000547

46 1 0.000000754 0.000004264 0.000001677

47 1 0.000001105 0.000002769 -0.000003212

-------------------------------------------------------------------

Zero-point correction= 0.352778 (Hartree/Particle)

Thermal correction to Energy= 0.379038

Thermal correction to Enthalpy= 0.379982

Thermal correction to Gibbs Free Energy= 0.289739

Sum of electronic and zero-point Energies= -1769.104171

Sum of electronic and thermal Energies= -1769.077912

Sum of electronic and thermal Enthalpies= -1769.076968

Sum of electronic and thermal Free Energies= -1769.167211

**Secondary amine**

-------------------------------------------------------------------

Center Atomic Forces (Hartrees/Bohr)

Number Number X Y Z

-------------------------------------------------------------------

1 6 -0.000003185 -0.000004295 -0.000024920

2 6 0.000004102 0.000005155 0.000013700

3 6 -0.000001905 -0.000014269 0.000026110

4 6 -0.000003387 0.000002979 0.000014172

5 6 0.000006714 -0.000000358 -0.000025641

6 1 0.000000724 -0.000010387 -0.000034390

7 1 -0.000001076 -0.000011067 0.000021533

8 1 -0.000002441 -0.000036628 0.000025764

9 1 -0.000000269 -0.000010247 0.000021752

10 1 0.000000438 0.000026730 -0.000033416

11 7 -0.000003959 -0.000014475 -0.000034821

12 1 0.000002232 0.000027814 0.000021232

13 1 -0.000001451 -0.000010467 -0.000034315

14 1 -0.000000091 -0.000005813 0.000056043

15 1 0.000001287 0.000027094 0.000021164

16 1 0.000002266 0.000028233 -0.000033967

-------------------------------------------------------------------

Zero-point correction= 0.141236 (Hartree/Particle)

Thermal correction to Energy= 0.147196

Thermal correction to Enthalpy= 0.148140

Thermal correction to Gibbs Free Energy= 0.110299

Sum of electronic and zero-point Energies= -251.280258

Sum of electronic and thermal Energies= -251.274299

Sum of electronic and thermal Enthalpies= -251.273354

Sum of electronic and thermal Free Energies= -251.311195

**Elimination of 4-methylbenzenesulfonate**

**Computation method**

The DFT calculations have been performed using the Gaussian 09 suite program that employed on the Aziz supercomputing facility in the High Performance Computing Centre of King Abdulaziz University (http://hpc.kau.edu.sa). The Becke’s three parameter exchange functional [1] and the Lee-Yang-Parr correlation functional [2] (B3LYP) method with the 6 311G+(d,p) basis set were used to the geometry optimizations, transition state and calculate the corresponding harmonic vibration frequency. The transition states have been characterized by a single imaginary frequency. The Gibbs free energies (ΔGsol) in this discussion have obtained in DMSO at 298 K and 1 atm and using the value of thermal corrections (Gcorr-gas) in the gas phase

**Molecular Dicking procedure**

Molecular operation environment software (MOE) has utilized to dock the complexes toward Human Leukocyte Elastase (PDB = 1EAT). We used the docking protocol that has been descried in our previous work [3]. After the crystal structure has downloaded from the PDB [www.rcsb.org](http://www.rcsb.org), the water molecules, co-ligand and the metal ions have been removed. The final structure obtained after 3D protonation and the correction process. The active binding sites were generated by the MOE site finder to create the dummy sites as binding pocket. The default docking parameters were as follows: triangle matcher for replacing the molecule and London dG for rescoring the docking scores. The DFT optimized structures of the sulfonic reactance and the products molecules have used to generate the best five binding poses with flexible molecules rotation. The hydrogen bonds formation between elastase and investigated compound were used to rank the binding affinity and presented as the free binding energy (S,kcal/mol). The higher negative values of the docking scores were presented along with 2D and 3D structures.

------------------------------------------------------------------

Center Atomic Forces (Hartrees/Bohr)

Number Number X Y Z

-------------------------------------------------------------------

1 6 -0.000008235 -0.000005554 0.000003577

2 6 0.000005335 0.000013360 -0.000002407

3 6 -0.000005745 -0.000006845 -0.000004759

4 6 -0.000007035 -0.000000762 -0.000000650

5 6 0.000009023 0.000005875 -0.000004704

6 6 -0.000001144 -0.000007058 0.000004664

7 1 0.000000641 0.000002611 -0.000000884

8 1 0.000003921 0.000000149 0.000003649

9 1 0.000001812 -0.000000062 0.000000632

10 1 -0.000001872 0.000001327 -0.000002838

11 16 0.000013847 -0.000008187 0.000004694

12 8 -0.000002798 -0.000001701 -0.000004454

13 8 -0.000005248 0.000002313 -0.000000344

14 8 -0.000001297 0.000004340 -0.000002982

15 6 -0.000002261 0.000000321 0.000010382

16 1 -0.000000917 -0.000001332 0.000000375

17 1 0.000000881 0.000000439 -0.000005169

18 1 0.000001092 0.000000766 0.000001219

-------------------------------------------------------------------

Zero-point correction= 0.129339 (Hartree/Particle)

Thermal correction to Energy= 0.139593

Thermal correction to Enthalpy= 0.140537

Thermal correction to Gibbs Free Energy= 0.090853

Sum of electronic and zero-point Energies= -894.885123

Sum of electronic and thermal Energies= -894.874869

Sum of electronic and thermal Enthalpies= -894.873925

Sum of electronic and thermal Free Energies= -894.923609

**TS1**

-------------------------------------------------------------------

Center Atomic Forces (Hartrees/Bohr)

Number Number X Y Z

-------------------------------------------------------------------

1 6 0.000000013 -0.000001309 0.000002794

2 6 -0.000000444 -0.000001268 0.000003259

3 6 -0.000000585 -0.000000821 0.000003692

4 6 -0.000000471 -0.000000488 0.000003430

5 6 -0.000001044 -0.000000468 -0.000000349

6 6 -0.000000048 -0.000000683 0.000002022

7 6 0.000000238 -0.000000980 0.000000751

8 8 0.000000325 -0.000000812 -0.000000309

9 6 0.000000132 0.000000036 0.000000113

10 8 -0.000000457 -0.000000839 0.000000956

11 7 0.000000292 0.000000824 -0.000000240

12 6 -0.000000751 0.000000803 -0.000000694

13 6 -0.000000195 0.000000844 -0.000000030

14 6 -0.000000225 0.000001094 -0.000002044

15 6 -0.000000392 0.000003408 -0.000001050

16 7 -0.000000050 0.000002883 0.000000168

17 8 -0.000000551 0.000002690 -0.000000429

18 8 0.000000343 0.000000718 -0.000002088

19 8 0.000000056 0.000001196 0.000000807

20 16 -0.000000897 0.000001890 0.000000450

21 8 -0.000002587 0.000002003 0.000001456

22 8 0.000001115 0.000001614 0.000000017

23 6 -0.000000073 -0.000000572 -0.000000234

24 6 -0.000000645 0.000001171 0.000000304

25 6 -0.000000290 0.000000394 0.000000571

26 6 -0.000000470 -0.000000059 0.000000510

27 6 -0.000000220 0.000000148 0.000001025

28 6 -0.000000230 0.000000447 0.000001253

29 6 -0.000000137 0.000000026 0.000000021

30 1 -0.000000041 -0.000001781 0.000002048

31 1 -0.000000395 -0.000001608 0.000003515

32 1 -0.000000724 -0.000000783 0.000004340

33 1 -0.000000723 -0.000000125 0.000003695

34 1 0.000000125 -0.000001356 0.000006808

35 1 -0.000001060 -0.000002363 0.000001782

36 1 -0.000000647 0.000000557 0.000000064

37 1 0.000000829 -0.000000704 0.000000097

38 1 -0.000000255 0.000001750 -0.000000913

39 1 0.000000106 0.000002087 -0.000001502

40 1 -0.000000326 0.000001923 0.000000235

41 1 -0.000000221 0.000001480 -0.000000445

42 1 -0.000000125 -0.000000572 0.000001278

43 1 -0.000000351 -0.000000236 0.000001866

44 1 -0.000000035 -0.000000668 0.000000050

45 1 -0.000000229 0.000000314 0.000000783

46 1 0.000000021 0.000000827 -0.000000513

47 1 -0.000000100 -0.000001560 0.000000577

48 6 0.000000490 -0.000000254 -0.000002742

49 6 0.000000708 -0.000000025 -0.000001459

50 6 0.000000843 -0.000000681 -0.000002600

51 6 0.000000891 -0.000001377 -0.000002378

52 6 0.000000866 -0.000001146 -0.000003005

53 1 0.000000841 0.000000389 -0.000003103

54 1 0.000000151 -0.000000421 -0.000001109

55 1 0.000000978 -0.000001021 -0.000001826

56 1 0.000000689 -0.000001846 -0.000001477

57 1 0.000000532 -0.000001325 -0.000003547

58 7 0.000000904 -0.000000260 -0.000003122

59 1 0.000000878 -0.000000064 -0.000003213

60 1 0.000000828 -0.000000760 -0.000003033

61 1 0.000001238 -0.000002185 -0.000002831

62 1 0.000000771 -0.000000689 -0.000002690

63 1 0.000000791 0.000000593 -0.000001764

-------------------------------------------------------------------

Ze ro-point correction= 0.497710 (Hartree/Particle)

Thermal correction to Energy= 0.532039

Thermal correction to Enthalpy= 0.532983

Thermal correction to Gibbs Free Energy= 0.423987

Sum of electronic and zero-point Energies= -2020.399157

Sum of electronic and thermal Energies= -2020.364829

Sum of electronic and thermal Enthalpies= -2020.363884

Sum of electronic and thermal Free Energies= -2020.472881

**R1-2**

-------------------------------------------------------------------

Center Atomic Forces (Hartrees/Bohr)

Number Number X Y Z

-------------------------------------------------------------------

1 6 0.000001039 0.000002013 0.000001241

2 6 -0.000000655 0.000002307 0.000000198

3 6 0.000000142 0.000002566 0.000001923

4 6 0.000001718 0.000003725 0.000002300

5 6 0.000000796 0.000002731 0.000001556

6 6 -0.000000184 -0.000000207 0.000003099

7 6 0.000001488 -0.000001365 0.000002880

8 8 0.000000299 -0.000000793 0.000003054

9 6 0.000002518 -0.000003049 0.000000698

10 8 -0.000001428 0.000004010 -0.000000423

11 7 0.000001166 0.000005167 -0.000001772

12 6 0.000002167 0.000000602 -0.000000521

13 6 -0.000002842 -0.000000380 0.000003498

14 6 -0.000001429 0.000001262 -0.000002589

15 6 0.000002862 0.000001687 0.000002397

16 7 -0.000005621 -0.000002586 -0.000000799

17 8 -0.000000788 0.000001916 0.000002297

18 8 -0.000000694 -0.000001803 -0.000000060

19 8 0.000003868 -0.000000551 -0.000003761

20 16 0.000001492 -0.000003421 0.000004986

21 8 -0.000000777 -0.000001811 -0.000001571

22 8 0.000001277 -0.000001822 0.000002161

23 6 0.000001285 0.000000962 -0.000000216

24 6 0.000000621 -0.000003212 -0.000000263

25 6 -0.000001109 -0.000003390 0.000000232

26 6 0.000000132 0.000000300 0.000000339

27 6 0.000001983 -0.000003032 0.000000971

28 6 -0.000000836 -0.000002608 0.000002752

29 6 0.000000027 -0.000002096 0.000000301

30 1 0.000000253 0.000000710 0.000000434

31 1 0.000000158 0.000002178 0.000000252

32 1 0.000000592 0.000003699 0.000001550

33 1 0.000001021 0.000003994 0.000002920

34 1 0.000000669 0.000002616 0.000003118

35 1 0.000000740 0.000001046 0.000002440

36 1 -0.000001237 -0.000000435 0.000002379

37 1 0.000001281 0.000000519 0.000001646

38 1 -0.000000249 -0.000002207 -0.000001197

39 1 -0.000000288 -0.000001914 -0.000001384

40 1 0.000000535 -0.000001843 0.000002232

41 1 0.000000912 -0.000002172 0.000002298

42 1 0.000000206 -0.000003068 0.000000583

43 1 0.000000501 -0.000001584 0.000001485

44 1 0.000000033 -0.000001489 -0.000000197

45 1 0.000000805 0.000000192 0.000001034

46 1 0.000000868 -0.000000580 0.000000218

47 6 -0.000001149 -0.000001922 -0.000000833

48 6 -0.000000618 0.000000239 -0.000002511

49 6 -0.000001845 0.000001509 -0.000004162

50 6 -0.000000548 -0.000000215 -0.000003534

51 6 -0.000001125 -0.000003399 -0.000001482

52 1 -0.000001169 0.000001728 -0.000002240

53 1 -0.000001226 0.000002211 -0.000002844

54 1 -0.000001437 0.000000937 -0.000004081

55 1 -0.000001678 -0.000001320 -0.000004219

56 1 -0.000000664 -0.000001622 -0.000002791

57 7 -0.000001674 0.000000627 -0.000003558

58 1 -0.000000990 -0.000000010 -0.000002553

59 1 -0.000000757 -0.000001279 -0.000002870

60 1 -0.000001067 0.000000243 -0.000004300

61 1 -0.000000653 0.000000923 -0.000002573

62 1 -0.000000116 0.000001893 -0.000001539

63 1 0.000001400 0.000002674 0.000001371

-------------------------------------------------------------------

Zero-point correction= 0.500111 (Hartree/Particle)

Thermal correction to Energy= 0.534247

Thermal correction to Enthalpy= 0.535191

Thermal correction to Gibbs Free Energy= 0.424354

Sum of electronic and zero-point Energies= -2020.508321

Sum of electronic and thermal Energies= -2020.474185

Sum of electronic and thermal Enthalpies= -2020.473241

Sum of electronic and thermal Free Energies= -2020.584078

**R1-4**

---------------------------------------------------------------------

Center Atomic Atomic Coordinates (Angstroms)

Number Number Type X Y Z

---------------------------------------------------------------------

1 6 0 8.874996 1.798242 -1.863255

2 6 0 10.182251 2.098524 -1.458486

3 6 0 10.776339 1.376133 -0.415002

4 6 0 10.063805 0.351531 0.221980

5 6 0 8.756807 0.050926 -0.182973

6 6 0 8.161069 0.776758 -1.222931

7 6 0 6.720813 0.453097 -1.659939

8 8 0 4.207027 0.597989 -2.460687

9 6 0 4.463891 0.957407 -1.282792

10 8 0 5.809472 1.247871 -0.896453

11 7 0 3.479668 1.071506 -0.385066

12 6 0 1.424288 2.079292 -1.279610

13 6 0 2.091706 0.786306 -0.774368

14 6 0 1.317545 0.255716 0.443752

15 6 0 -0.748874 -0.992759 0.697457

16 7 0 -0.072615 -0.028064 0.063948

17 8 0 -1.389063 -1.901891 1.292971

18 8 0 1.488077 2.386283 -2.499196

19 1 0 8.421329 2.349110 -2.660356

20 1 0 10.726875 2.879507 -1.946093

21 1 0 11.773543 1.607186 -0.104454

22 1 0 10.518191 -0.201300 1.017402

23 1 0 8.213017 -0.732029 0.302752

24 1 0 6.519865 -0.583982 -1.491507

25 1 0 3.687870 1.350140 0.552032

26 1 0 1.729623 0.114488 1.421068

27 1 0 6.603623 0.673516 -2.700641

28 1 0 2.083329 0.054156 -1.552835

29 6 0 0.160140 2.126145 0.753080

30 6 0 -0.586391 3.017355 1.761763

31 6 0 -1.727414 3.740855 1.038689

32 6 0 -1.129891 4.582983 -0.092873

33 6 0 -0.369875 3.654997 -1.058556

34 1 0 -0.736864 1.930302 0.203599

35 1 0 -0.984132 2.414718 2.550849

36 1 0 -2.407335 3.021742 0.631072

37 1 0 -1.912512 5.089094 -0.618972

38 1 0 0.049935 4.241744 -1.849474

39 7 0 0.711859 2.952697 -0.338910

40 1 0 -1.044477 2.934979 -1.466775

41 1 0 -0.454053 5.304527 0.315993

42 1 0 -2.251607 4.371936 1.724352

43 1 0 0.090278 3.737661 2.172120

44 1 0 1.070147 2.189010 1.312397

45 1 0 1.618754 -0.751773 0.245916

---------------------------------------------------------------------

Zero-point correction= 0.369452 (Hartree/Particle)

Thermal correction to Energy= 0.392581

Thermal correction to Enthalpy= 0.393525

Thermal correction to Gibbs Free Energy= 0.310766

Sum of electronic and zero-point Energies= -1125.610692

Sum of electronic and thermal Energies= -1125.587563

Sum of electronic and thermal Enthalpies= -1125.586619

Sum of electronic and thermal Free Energies= -1125.669378

**TS3**

---------------------------------------------------------------------

Center Atomic Atomic Coordinates (Angstroms)

Number Number Type X Y Z

---------------------------------------------------------------------

1 6 0 8.129130 2.264348 0.032717

2 6 0 9.335282 1.745776 -0.438980

3 6 0 9.870858 2.205811 -1.641815

4 6 0 9.199553 3.190258 -2.368814

5 6 0 7.996849 3.709497 -1.893375

6 6 0 7.447342 3.248800 -0.690762

7 6 0 6.135530 3.803085 -0.195487

8 8 0 4.726691 1.691708 0.816986

9 6 0 4.409005 2.124106 -0.278446

10 8 0 5.005363 3.188498 -0.879627

11 7 0 3.412173 1.663629 -1.075705

12 6 0 1.053045 0.830629 -0.760286

13 6 0 2.560488 0.478880 -0.918860

14 6 0 3.070690 -0.577614 0.090841

15 6 0 2.582677 -2.867812 -0.622495

16 7 0 2.241706 -1.767197 0.038382

17 8 0 3.528482 -3.223743 -1.310175

18 8 0 0.294204 0.430413 -1.652506

19 1 0 7.713812 1.904531 0.967482

20 1 0 9.855884 0.985389 0.132268

21 1 0 10.808528 1.803756 -2.008800

22 1 0 9.615625 3.556432 -3.300660

23 1 0 7.482276 4.480771 -2.457320

24 1 0 6.043216 4.862642 -0.431037

25 1 0 3.314737 2.151185 -1.955058

26 1 0 3.076094 -0.197208 1.109068

27 1 0 6.019274 3.656049 0.876658

28 1 0 2.571216 -0.015835 -1.891134

29 6 0 1.408831 2.059783 1.418242

30 6 0 1.260209 3.581936 1.535990

31 6 0 -0.213717 3.991828 1.646616

32 6 0 -1.033868 3.384601 0.501020

33 6 0 -0.824887 1.868841 0.411752

34 1 0 1.048281 1.582792 2.338898

35 1 0 1.823149 3.922850 2.410065

36 1 0 -0.615478 3.640538 2.605414

37 1 0 -2.100364 3.587199 0.638220

38 1 0 -1.335725 1.444504 -0.448131

39 7 0 0.606138 1.541229 0.302520

40 1 0 -1.213616 1.383722 1.315993

41 1 0 -0.738260 3.840292 -0.450981

42 1 0 -0.306198 5.081752 1.646329

43 1 0 1.712208 4.051512 0.655071

44 1 0 2.451005 1.787117 1.301817

45 1 0 4.102491 -0.828707 -0.172518

46 6 0 0.465524 -4.043485 -1.398571

47 6 0 0.962843 -5.248782 -2.201641

48 6 0 1.204796 -6.457308 -1.287503

49 6 0 2.151131 -6.084792 -0.138657

50 6 0 1.630499 -4.864225 0.626001

51 1 0 -0.518357 -4.257027 -0.968661

52 1 0 0.219493 -5.484697 -2.968840

53 1 0 0.247675 -6.800548 -0.874840

54 1 0 2.254559 -6.916517 0.564466

55 1 0 2.334095 -4.538564 1.394963

56 1 0 1.206162 -2.523012 0.319416

57 7 0 1.356849 -3.699116 -0.258253

58 1 0 0.684256 -5.105374 1.120870

59 1 0 3.147850 -5.867827 -0.533813

60 1 0 1.617846 -7.290412 -1.862916

61 1 0 1.889721 -4.983317 -2.718622

62 1 0 0.368112 -3.153989 -2.024111

---------------------------------------------------------------------

Zero-point correction= 0.527375 (Hartree/Particle)

Thermal correction to Energy= 0.555917

Thermal correction to Enthalpy= 0.556861

Thermal correction to Gibbs Free Energy= 0.462305

Sum of electronic and zero-point Energies= -1377.391306

Sum of electronic and thermal Energies= -1377.362764

Sum of electronic and thermal Enthalpies= -1377.361820

Sum of electronic and thermal Free Energies= -1377.456376

**R1-6**

-------------------------------------------------------------------

Center Atomic Forces (Hartrees/Bohr)

Number Number X Y Z

-------------------------------------------------------------------

1 6 -0.000000810 0.000004169 -0.000000087

2 6 -0.000004720 0.000002536 -0.000003584

3 6 -0.000002401 0.000003733 -0.000002295

4 6 -0.000002413 0.000005403 0.000001820

5 6 -0.000003187 0.000004397 -0.000000690

6 6 -0.000001048 0.000000405 -0.000001943

7 6 0.000000226 0.000000420 0.000002258

8 8 0.000004397 -0.000002444 0.000002979

9 6 -0.000005539 0.000000128 -0.000002583

10 8 0.000000864 0.000000891 -0.000000836

11 7 -0.000006379 0.000010198 -0.000001500

12 6 0.000001610 -0.000001468 0.000001382

13 6 0.000004014 -0.000005543 0.000003239

14 6 -0.000003264 -0.000010316 -0.000002248

15 6 -0.000003807 0.000011045 0.000010796

16 7 0.000004367 0.000004878 0.000007216

17 8 0.000000572 -0.000001671 -0.000009294

18 8 0.000002236 -0.000006433 -0.000003851

19 1 -0.000003157 0.000002506 -0.000004263

20 1 -0.000003732 0.000005285 -0.000002806

21 1 -0.000003345 0.000006504 -0.000000528

22 1 -0.000001492 0.000003964 0.000001063

23 1 0.000000018 0.000001809 0.000001349

24 1 0.000000984 -0.000000245 -0.000001830

25 1 0.000002827 -0.000001548 0.000000854

26 1 0.000000229 0.000001884 0.000004103

27 1 -0.000001585 -0.000000367 -0.000002854

28 1 -0.000001541 -0.000003368 0.000001423

29 6 -0.000000831 0.000000744 -0.000004644

30 6 -0.000005815 -0.000002371 -0.000004574

31 6 0.000002011 0.000000833 -0.000008250

32 6 -0.000000747 -0.000005756 -0.000000876

33 6 -0.000001212 0.000001371 -0.000003631

34 1 -0.000001249 0.000000436 0.000000397

35 1 -0.000001922 0.000002404 -0.000002871

36 1 -0.000003735 -0.000000445 -0.000003706

37 1 -0.000001889 -0.000002016 -0.000006894

38 1 0.000000931 -0.000003286 -0.000002991

39 7 -0.000002127 0.000009326 -0.000000931

40 1 -0.000002019 -0.000003137 -0.000003600

41 1 -0.000000594 -0.000001135 -0.000006373

42 1 -0.000003353 0.000000898 -0.000005451

43 1 -0.000000792 0.000001912 -0.000003446

44 1 0.000000515 0.000001855 -0.000001244

45 1 0.000002679 0.000002691 0.000004189

46 6 0.000000993 0.000001994 0.000001601

47 6 0.000004016 -0.000002119 0.000003030

48 6 0.000003337 0.000000223 0.000003137

49 6 0.000005205 -0.000004912 0.000007506

50 6 -0.000001341 -0.000003411 0.000002764

51 1 0.000001592 -0.000000419 0.000004282

52 1 0.000001449 -0.000001629 0.000003695

53 1 0.000003975 -0.000004416 0.000005889

54 1 0.000003695 -0.000004682 0.000002558

55 1 0.000003355 -0.000003100 0.000002497

56 1 -0.000001685 -0.000000027 0.000001569

57 7 0.000012188 -0.000008729 0.000006237

58 1 0.000001749 -0.000001386 0.000004889

59 1 0.000002365 -0.000002935 0.000000893

60 1 0.000002870 -0.000004696 0.000002196

61 1 0.000000048 -0.000001684 0.000001673

62 1 0.000002415 0.000000854 0.000003187

-------------------------------------------------------------------

Zero-point correction= 0.531511 (Hartree/Particle)

Thermal correction to Energy= 0.560879

Thermal correction to Enthalpy= 0.561823

Thermal correction to Gibbs Free Energy= 0.463628

Sum of electronic and zero-point Energies= -1377.463527

Sum of electronic and thermal Energies= -1377.434159

Sum of electronic and thermal Enthalpies= -1377.433215

Sum of electronic and thermal Free Energies= -1377.531411

**R2**

**TS1**

-------------------------------------------------------------------

Center Atomic Forces (Hartrees/Bohr)

Number Number X Y Z

-------------------------------------------------------------------

1 6 -0.000002027 0.000001321 -0.000015932

2 6 0.000000871 0.000005938 -0.000013893

3 6 0.000005280 0.000006385 -0.000012467

4 6 0.000007007 0.000007017 -0.000006913

5 6 0.000007509 0.000017398 -0.000001149

6 6 0.000002480 0.000007038 -0.000003467

7 6 -0.000001290 0.000000685 -0.000006262

8 8 -0.000004526 -0.000000135 -0.000002770

9 6 0.000001410 0.000000420 -0.000002834

10 8 -0.000002208 0.000004942 -0.000009632

11 7 0.000010915 0.000017317 -0.000002711

12 6 -0.000008365 -0.000001372 -0.000005907

13 6 -0.000002997 0.000005120 -0.000004216

14 6 -0.000000225 0.000005275 -0.000001698

15 6 -0.000002533 0.000020745 -0.000018357

16 7 0.000017983 0.000004203 0.000000451

17 8 0.000015234 -0.000004934 0.000006509

18 8 -0.000000356 0.000002956 -0.000004233

19 8 0.000012054 0.000007950 -0.000008205

20 16 -0.000003779 0.000034434 -0.000041783

21 8 0.000042189 -0.000037074 0.000020995

22 8 -0.000009317 -0.000018795 0.000025631

23 6 -0.000008165 0.000002772 0.000032244

24 6 0.000004760 -0.000003272 0.000007395

25 6 0.000012315 0.000005154 0.000010287

26 6 -0.000002272 -0.000006997 0.000014460

27 6 -0.000014677 -0.000019237 0.000011233

28 6 0.000001530 0.000000861 0.000007140

29 6 -0.000005751 -0.000008677 0.000006405

30 1 -0.000002961 0.000006357 -0.000010896

31 1 0.000000122 0.000007392 -0.000016620

32 1 0.000006778 0.000007618 -0.000014219

33 1 0.000009705 0.000004434 -0.000008891

34 1 0.000016529 -0.000010338 -0.000016816

35 1 -0.000000847 0.000001635 -0.000007503

36 1 -0.000000567 -0.000005181 -0.000007383

37 1 0.000007323 -0.000008400 0.000004708

38 1 0.000009622 -0.000001182 -0.000004917

39 1 0.000011452 0.000005700 0.000000827

40 1 0.000007141 -0.000003476 0.000008946

41 1 0.000003055 -0.000006660 0.000012306

42 1 -0.000006090 -0.000006211 0.000010230

43 1 -0.000000710 -0.000003608 0.000006017

44 1 -0.000014150 -0.000002452 0.000027349

45 1 0.000003913 -0.000002435 0.000007625

46 1 -0.000010193 -0.000020782 0.000012506

47 1 -0.000001208 0.000002346 -0.000005896

48 6 -0.000008932 -0.000002050 -0.000002068

49 6 -0.000000148 0.000004376 0.000005199

50 6 -0.000000655 -0.000004504 0.000006104

51 6 -0.000010010 -0.000002976 0.000000737

52 6 -0.000014454 0.000000390 -0.000002761

53 1 -0.000007516 -0.000003652 0.000002595

54 1 -0.000005032 -0.000002272 0.000004527

55 1 -0.000004177 -0.000000218 0.000008094

56 1 -0.000008848 -0.000000413 -0.000003378

57 1 -0.000009551 -0.000000755 -0.000002157

58 7 0.000001799 0.000001310 -0.000002291

59 1 -0.000006291 0.000001055 -0.000003573

60 1 -0.000010681 -0.000003523 -0.000002604

61 1 -0.000017450 -0.000001767 0.000000562

62 1 -0.000008982 -0.000003521 0.000006746

63 1 -0.000001035 -0.000003674 0.000006574

-------------------------------------------------------------------

Zero-point correction= 0.506320 (Hartree/Particle)

Thermal correction to Energy= 0.540170

Thermal correction to Enthalpy= 0.541114

Thermal correction to Gibbs Free Energy= 0.437898

Sum of electronic and zero-point Energies= -2020.851032

Sum of electronic and thermal Energies= -2020.817182

Sum of electronic and thermal Enthalpies= -2020.816237

Sum of electronic and thermal Free Energies= -2020.919454

**R2-2**

---------------------------------------------------------------------

Center Atomic Atomic Coordinates (Angstroms)

Number Number Type X Y Z

---------------------------------------------------------------------

1 6 0 6.539084 1.875329 -2.118003

2 6 0 7.490643 2.884734 -2.257672

3 6 0 8.291690 3.243968 -1.173034

4 6 0 8.138621 2.589396 0.049570

5 6 0 7.185549 1.580814 0.186076

6 6 0 6.375964 1.215942 -0.894853

7 6 0 5.335874 0.141190 -0.739709

8 8 0 3.074991 -1.255103 -0.311058

9 6 0 3.030338 -0.043789 -0.145837

10 8 0 4.094051 0.781570 -0.332245

11 7 0 1.948438 0.656387 0.258705

12 6 0 -0.379318 1.173152 -0.110065

13 6 0 0.617565 0.076378 0.354948

14 6 0 0.289437 -0.491338 1.763778

15 6 0 0.071497 0.534384 2.874334

16 7 0 -1.445136 0.605539 -0.651602

17 8 0 -1.068796 0.988086 3.044551

18 8 0 -0.084287 2.375724 0.070956

19 8 0 -2.427695 1.698348 -0.955834

20 16 0 -3.103738 1.479950 -2.412158

21 8 0 -2.094165 1.626106 -3.466202

22 8 0 -3.913205 0.256957 -2.417489

23 6 0 -4.174247 2.907056 -2.397223

24 6 0 -3.713225 4.111959 -2.924675

25 6 0 -4.550228 5.222749 -2.895406

26 6 0 -5.837698 5.147235 -2.347424

27 6 0 -6.269922 3.921971 -1.822157

28 6 0 -5.448606 2.799433 -1.842854

29 6 0 -6.747636 6.348687 -2.350333

30 1 0 5.921444 1.594460 -2.964793

31 1 0 7.609811 3.386580 -3.211270

32 1 0 9.034424 4.026316 -1.281491

33 1 0 8.762449 2.861045 0.893644

34 1 0 7.071216 1.070711 1.136895

35 1 0 5.622313 -0.583631 0.023695

36 1 0 1.980536 1.669114 0.254954

37 1 0 -0.657443 -1.025567 1.678905

38 1 0 1.053913 -1.225436 2.018979

39 1 0 -2.721099 4.178572 -3.352439

40 1 0 -4.196274 6.162382 -3.304803

41 1 0 -7.261960 3.843514 -1.391438

42 1 0 -5.793234 1.855672 -1.439869

43 1 0 -7.343028 6.374839 -3.269290

44 1 0 -7.443803 6.322350 -1.509858

45 1 0 -6.177902 7.278688 -2.302590

46 1 0 5.164044 -0.387214 -1.678531

47 1 0 0.562709 -0.768865 -0.331428

48 6 0 2.480123 0.371403 3.626318

49 6 0 3.525565 1.493270 3.599343

50 6 0 3.322273 2.478930 4.755058

51 6 0 1.880205 2.998036 4.766618

52 6 0 0.877326 1.840856 4.781535

53 1 0 2.627011 -0.245332 4.523584

54 1 0 4.521214 1.041768 3.644851

55 1 0 3.533580 1.973872 5.706085

56 1 0 1.701619 3.630366 5.641612

57 1 0 -0.146178 2.197893 4.709248

58 7 0 1.114883 0.915587 3.662053

59 1 0 0.975947 1.277527 5.719533

60 1 0 1.699962 3.615047 3.878881

61 1 0 4.029004 3.309930 4.674300

62 1 0 3.453098 2.022145 2.643235

63 1 0 2.601392 -0.270577 2.760721

---------------------------------------------------------------------

Zero-point correction= 0.500444 (Hartree/Particle)

Thermal correction to Energy= 0.533352

Thermal correction to Enthalpy= 0.534296

Thermal correction to Gibbs Free Energy= 0.428795

Sum of electronic and zero-point Energies= -2020.518179

Sum of electronic and thermal Energies= -2020.485271

Sum of electronic and thermal Enthalpies= -2020.484327

Sum of electronic and thermal Free Energies= -2020.589827

**R2-3**

-------------------------------------------------------------------

Center Atomic Forces (Hartrees/Bohr)

Number Number X Y Z

-------------------------------------------------------------------

1 6 -0.000067337 -0.000208624 -0.000013213

2 6 0.000026136 -0.000086230 0.000102302

3 6 0.000200034 0.000020752 -0.000001773

4 6 0.000067005 -0.000087590 -0.000110877

5 6 -0.000099610 0.000166909 -0.000188377

6 6 -0.000147486 0.000173795 0.000216531

7 6 0.000126075 0.000447265 0.000038127

8 8 -0.001268639 -0.001837876 -0.001163023

9 6 -0.000540283 0.003342387 0.001681478

10 8 0.001325501 -0.001046235 -0.000352361

11 7 0.001549069 -0.000723751 0.000018057

12 6 0.003394077 -0.045018749 0.031471014

13 6 -0.024577404 0.013665084 -0.029491399

14 6 0.056079618 -0.018584545 0.025302623

15 6 -0.017846815 0.015934032 -0.010562611

16 7 0.122377085 0.001587855 0.020602693

17 8 -0.004130931 -0.003742279 0.002666669

18 8 0.005558951 0.024593762 -0.018366707

19 8 -0.104526224 0.007856874 -0.027217489

20 16 -0.014124895 0.002057138 -0.005454846

21 8 -0.003590576 -0.001335630 0.003566451

22 8 0.000208850 0.000166066 -0.000728838

23 6 -0.005327882 0.002059792 0.001225734

24 6 0.000196501 0.000324103 -0.000277063

25 6 0.000406792 0.000180332 0.000408770

26 6 -0.000081334 0.000424512 0.000487843

27 6 -0.000529797 0.000883424 -0.000513910

28 6 0.000535923 0.000100887 -0.002136113

29 6 -0.000334105 0.000240972 0.000087331

30 1 -0.000037900 0.000013099 0.000046771

31 1 -0.000016290 0.000041560 -0.000000945

32 1 -0.000000183 0.000016579 0.000014312

33 1 -0.000021169 0.000048481 -0.000004613

34 1 0.000078313 -0.000039236 0.000230268

35 1 0.000032211 -0.000146221 -0.000256135

36 1 0.000487108 0.000023324 -0.001114951

37 1 -0.028002022 0.012664235 -0.022284072

38 1 0.014459558 -0.016622968 0.027361278

39 1 -0.000408765 -0.000575214 0.000114343

40 1 0.000038405 0.000010842 -0.000094463

41 1 -0.000283629 0.000143538 -0.000265247

42 1 -0.000217810 -0.000424587 -0.000036957

43 1 -0.000057830 -0.000240984 -0.000122734

44 1 0.000088692 -0.000093092 0.000077979

45 1 -0.000232996 -0.000073105 -0.000156134

46 1 -0.000002263 0.000047819 -0.000050424

47 1 -0.000494166 -0.001457054 0.000151954

48 6 -0.000064356 -0.000936716 0.001357173

49 6 -0.000119877 0.000369281 -0.000296151

50 6 -0.000027132 -0.000147665 0.000447869

51 6 -0.000449318 0.000714085 -0.000445783

52 6 0.000199254 0.001808420 0.001047334

53 1 0.000258802 0.000372639 0.000137255

54 1 -0.000054511 0.000046638 -0.000018349

55 1 0.000125045 -0.000178447 -0.000068565

56 1 0.000041896 0.000264562 0.000283264

57 1 0.000068396 0.002265462 0.001061919

58 7 -0.000116757 0.001769097 0.001798836

59 1 -0.000492073 -0.000256257 -0.000218784

60 1 0.000378311 -0.000594217 0.000230228

61 1 0.000035532 -0.000012847 0.000044776

62 1 -0.000021293 -0.000254638 0.000033352

63 1 -0.000029480 -0.000120846 -0.000301627

-------------------------------------------------------------------

Zero-point correction= 0.499570 (Hartree/Particle)

Thermal correction to Energy= 0.534041

Thermal correction to Enthalpy= 0.534985

Thermal correction to Gibbs Free Energy= 0.420901

Sum of electronic and zero-point Energies= -2020.608116

Sum of electronic and thermal Energies= -2020.573644

Sum of electronic and thermal Enthalpies= -2020.572700

Sum of electronic and thermal Free Energies= -2020.686784

**R2-4**

-------------------------------------------------------------------

Center Atomic Forces (Hartrees/Bohr)

Number Number X Y Z

-------------------------------------------------------------------

1 6 0.000000538 -0.000001050 -0.000000462

2 6 0.000000341 -0.000001445 -0.000000907

3 6 0.000000608 -0.000001014 0.000000058

4 6 0.000000409 0.000000005 -0.000000281

5 6 -0.000000638 -0.000000830 0.000000058

6 6 0.000000950 -0.000001113 0.000000792

7 6 -0.000002635 -0.000000146 -0.000000134

8 8 0.000001191 -0.000003731 0.000000548

9 6 -0.000005946 0.000004060 0.000002985

10 8 0.000003312 -0.000002123 0.000002471

11 7 0.000004874 0.000001809 -0.000003023

12 6 -0.000002128 -0.000001413 0.000000443

13 6 -0.000005564 -0.000001484 0.000000957

14 6 0.000001000 0.000000565 0.000001335

15 6 0.000001017 0.000000030 -0.000000635

16 7 0.000002665 -0.000000246 0.000005031

17 8 -0.000000735 -0.000000144 -0.000000763

18 8 0.000001753 -0.000002124 -0.000000991

19 1 0.000000469 -0.000001926 0.000000108

20 1 0.000000904 -0.000001707 -0.000000696

21 1 0.000000446 -0.000000939 -0.000000953

22 1 -0.000000019 -0.000000307 -0.000000031

23 1 0.000000094 -0.000000057 0.000000746

24 1 0.000000198 -0.000000603 0.000001597

25 1 0.000000258 -0.000002095 -0.000000130

26 1 -0.000001335 -0.000000091 0.000001113

27 1 0.000000014 0.000000335 -0.000000396

28 1 0.000000387 -0.000001637 0.000001349

29 1 0.000000223 0.000000216 0.000000949

30 6 0.000000067 0.000000345 0.000001982

31 6 0.000000486 0.000002036 0.000001012

32 6 -0.000000026 0.000000650 -0.000001205

33 6 -0.000000272 0.000000876 -0.000001321

34 6 0.000000669 0.000001318 -0.000001120

35 1 -0.000000070 0.000001607 -0.000001407

36 1 -0.000000363 0.000001768 -0.000000430

37 1 -0.000000045 0.000001732 -0.000001383

38 1 0.000000389 0.000000855 -0.000001799

39 1 0.000000362 0.000000594 -0.000000789

40 7 -0.000002772 0.000002881 -0.000000491

41 1 -0.000000559 0.000001455 -0.000001104

42 1 0.000000211 0.000000381 -0.000001314

43 1 0.000000043 0.000001302 -0.000001382

44 1 -0.000000495 0.000000467 -0.000000727

45 1 -0.000000277 0.000000938 0.000000338

-------------------------------------------------------------------

Zero-point correction= 0.369617 (Hartree/Particle)

Thermal correction to Energy= 0.392421

Thermal correction to Enthalpy= 0.393365

Thermal correction to Gibbs Free Energy= 0.311644

Sum of electronic and zero-point Energies= -1125.617967

Sum of electronic and thermal Energies= -1125.595163

Sum of electronic and thermal Enthalpies= -1125.594219

Sum of electronic and thermal Free Energies= -1125.675940

**R2-6**

---------------------------------------------------------------------

Center Atomic Atomic Coordinates (Angstroms)

Number Number Type X Y Z

---------------------------------------------------------------------

1 6 0 8.817121 1.648709 -1.189617

2 6 0 9.581456 2.815075 -1.190192

3 6 0 9.803094 3.504761 0.002072

4 6 0 9.260711 3.022507 1.194030

5 6 0 8.498057 1.855680 1.191398

6 6 0 8.266628 1.159836 -0.000283

7 6 0 7.423164 -0.084787 -0.003201

8 8 0 5.388143 -1.848407 0.039335

9 6 0 5.110415 -0.671104 -0.132207

10 8 0 6.035701 0.322697 -0.175290

11 7 0 3.867624 -0.162457 -0.306525

12 6 0 2.004218 -1.645904 -2.543916

13 6 0 2.649614 -0.971271 -0.258574

14 6 0 1.827478 -0.673016 1.004979

15 6 0 1.329017 0.771115 1.074676

16 7 0 1.843697 -0.813085 -1.453800

17 8 0 1.565280 1.548986 0.134340

18 8 0 2.653685 -2.698711 -2.459568

19 1 0 8.649173 1.112479 -2.117824

20 1 0 10.005393 3.182539 -2.117902

21 1 0 10.399538 4.410141 0.003518

22 1 0 9.435239 3.551736 2.124006

23 1 0 8.081795 1.480347 2.120542

24 1 0 7.512048 -0.630772 0.936772

25 1 0 3.756361 0.840711 -0.356841

26 1 0 0.976313 -1.358887 1.035749

27 1 0 2.448144 -0.894394 1.876899

28 1 0 7.694958 -0.750471 -0.823766

29 1 0 2.974093 -2.007995 -0.223676

30 6 0 0.306127 0.289563 3.314488

31 6 0 0.826039 0.893706 4.624675

32 6 0 0.323857 2.330897 4.810647

33 6 0 0.652072 3.185874 3.579874

34 6 0 0.135308 2.530441 2.294819

35 1 0 -0.785993 0.192653 3.357721

36 1 0 0.503867 0.259747 5.455998

37 1 0 -0.762714 2.317587 4.963073

38 1 0 0.209223 4.182124 3.671329

39 1 0 0.444227 3.081602 1.410617

40 7 0 0.639172 1.153796 2.172496

41 1 0 -0.961138 2.491270 2.308887

42 1 0 1.736767 3.320141 3.498910

43 1 0 0.763203 2.772529 5.709613

44 1 0 1.921805 0.879939 4.611567

45 1 0 0.713554 -0.705384 3.161416

46 6 0 0.109741 0.227182 -5.225041

47 6 0 1.274729 0.025504 -6.199543

48 6 0 1.812935 -1.426627 -6.130635

49 6 0 1.350819 -2.147006 -4.853950

50 6 0 0.565800 -0.022307 -3.782104

51 1 0 -0.280798 1.246017 -5.286603

52 1 0 0.963636 0.271769 -7.217693

53 1 0 1.465237 -2.007805 -6.990007

54 1 0 1.989405 -2.996761 -4.628445

55 1 0 1.441076 0.105423 -1.569289

56 1 0 -0.302358 -0.087070 -3.114439

57 7 0 1.380001 -1.248394 -3.694918

58 1 0 -0.718063 -0.441251 -5.481424

59 1 0 2.072034 0.729790 -5.939056

60 1 0 2.905539 -1.421502 -6.173063

61 1 0 0.331573 -2.530186 -4.982977

62 1 0 1.172650 0.832705 -3.463077

---------------------------------------------------------------------

Zero-point correction= 0.531833 (Hartree/Particle)

Thermal correction to Energy= 0.560922

Thermal correction to Enthalpy= 0.561866

Thermal correction to Gibbs Free Energy= 0.465376

Sum of electronic and zero-point Energies= -1377.472981

Sum of electronic and thermal Energies= -1377.443892

Sum of electronic and thermal Enthalpies= -1377.442948

Sum of electronic and thermal Free Energies= -1377.539437

**R3____TS1**

-------------------------------------------------------------------

Center Atomic Forces (Hartrees/Bohr)

Number Number X Y Z

-------------------------------------------------------------------

1 6 0.000014603 -0.000005919 0.000018462

2 6 -0.000018052 0.000008146 -0.000004609

3 6 -0.000018294 -0.000007232 0.000011078

4 6 0.000005021 -0.000005888 -0.000043115

5 6 0.000026844 0.000098225 0.000010500

6 6 0.000043158 -0.000081429 0.000004141

7 6 -0.000158382 0.000023290 0.000046295

8 8 0.000013865 0.000373279 0.000124673

9 6 0.000094366 0.000370298 0.000263872

10 8 0.000242226 -0.000705702 -0.000373296

11 7 -0.000531584 0.000106835 -0.000352330

12 6 -0.000332530 -0.000250418 -0.000494203

13 6 0.001190856 -0.000419685 0.000387466

14 6 -0.000754724 0.000537329 0.000139651

15 6 0.000292646 0.000603368 0.000919025

16 7 0.000769633 -0.000020137 0.000442976

17 8 -0.000160904 -0.000850274 -0.001331861

18 8 -0.000254379 0.000643061 0.000405631

19 8 -0.000006886 -0.000293162 0.000000795

20 16 -0.000289936 0.000320096 0.000030171

21 8 -0.000189413 -0.000060755 -0.000110288

22 8 0.000159016 -0.000085796 0.000061580

23 6 0.001342812 0.000857051 0.001937659

24 6 -0.001578165 0.001867586 -0.000699346

25 6 0.000339974 -0.001045610 -0.001527274

26 6 0.001881448 0.000266049 0.005254076

27 6 -0.001342702 0.000931479 -0.000815405

28 6 0.001278539 -0.001843701 -0.001829551

29 6 -0.001427119 -0.001826122 -0.000241716

30 1 -0.000003571 -0.000041335 0.000042092

31 1 0.000003359 0.000005347 0.000000151

32 1 0.000000798 0.000002066 -0.000003122

33 1 0.000000481 0.000002025 0.000001317

34 1 0.000003345 -0.000016072 0.000002461

35 1 -0.000013895 -0.000038952 -0.000047396

36 1 0.000046455 0.000104793 0.000054230

37 1 -0.000133259 -0.000055133 -0.000090898

38 1 -0.000087920 0.000002682 0.000017354

39 1 -0.000104799 -0.000142044 0.000079492

40 1 -0.000608384 -0.000390458 0.000039648

41 1 -0.000554480 -0.000517174 0.000027506

42 1 -0.000025589 -0.000082425 0.000091207

43 1 0.000742736 0.000242524 -0.001024107

44 1 0.000813678 0.000448022 -0.000824958

45 1 -0.000598926 0.001240174 -0.000522552

46 1 0.000050645 -0.000042104 -0.000084702

47 1 0.000106282 0.000091887 0.000400308

48 6 -0.000055608 0.000015488 -0.000043986

49 6 0.000046440 0.000096161 -0.000021174

50 6 -0.000388783 0.000161843 -0.000081588

51 6 -0.000011833 -0.000071214 -0.000167187

52 6 -0.000111209 0.000016563 -0.000047275

53 1 0.000047934 0.000004562 -0.000036300

54 1 -0.000027710 0.000022118 -0.000006155

55 7 0.000267576 -0.000551170 0.000006689

56 1 -0.000018941 0.000020881 -0.000011698

57 1 0.000035089 -0.000031930 -0.000023449

58 1 -0.000065465 0.000027978 0.000019668

59 1 -0.000001300 -0.000046891 -0.000024034

60 1 -0.000015525 0.000004596 -0.000007687

61 1 -0.000005333 0.000010331 0.000028412

62 1 0.000017920 0.000008975 0.000002272

63 1 0.000017850 -0.000006372 0.000020399

-------------------------------------------------------------------

Zero-point correction= 0.496529 (Hartree/Particle)

Thermal correction to Energy= 0.530603

Thermal correction to Enthalpy= 0.531547

Thermal correction to Gibbs Free Energy= 0.421473

Sum of electronic and zero-point Energies= -2020.368196

Sum of electronic and thermal Energies= -2020.334123

Sum of electronic and thermal Enthalpies= -2020.333178

Sum of electronic and thermal Free Energies= -2020.443253

**R3**

------------------------------------------------------------------

Center Atomic Forces (Hartrees/Bohr)

Number Number X Y Z

-------------------------------------------------------------------

1 6 -0.000000091 -0.000001737 0.000001997

2 6 -0.000001269 -0.000000617 0.000001698

3 6 0.000000606 0.000000379 0.000000332

4 6 0.000000644 -0.000001553 -0.000002224

5 6 -0.000001046 -0.000000069 -0.000001688

6 6 0.000001235 0.000001666 -0.000000061

7 6 -0.000000117 0.000000044 0.000002216

8 8 -0.000000148 0.000003400 0.000005662

9 6 0.000000321 0.000002513 -0.000002562

10 8 0.000001038 0.000000995 -0.000001177

11 7 -0.000000078 0.000000269 0.000000858

12 6 -0.000000876 -0.000003669 0.000002060

13 6 0.000000162 0.000000049 0.000001407

14 6 0.000000435 0.000002580 0.000000787

15 6 0.000005232 -0.000002169 0.000001028

16 7 -0.000000866 0.000009313 0.000002182

17 8 -0.000004145 0.000004409 0.000002697

18 8 0.000000369 -0.000002093 -0.000004205

19 8 -0.000001786 -0.000007960 -0.000002457

20 16 0.000007359 0.000006804 0.000000100

21 8 -0.000005250 -0.000003389 -0.000008047

22 8 -0.000000180 -0.000007960 -0.000007370

23 6 0.000002426 0.000021503 0.000009414

24 6 -0.000003176 -0.000015694 0.000006003

25 6 -0.000003478 0.000010504 -0.000002648

26 6 -0.000000283 -0.000009152 -0.000004611

27 6 0.000001747 0.000001209 0.000000961

28 6 -0.000001132 -0.000013679 -0.000003952

29 6 0.000001486 0.000002508 0.000000976

30 1 -0.000000051 -0.000000793 0.000003571

31 1 -0.000000121 -0.000000726 0.000003320

32 1 -0.000000080 -0.000000598 0.000000172

33 1 -0.000000175 -0.000000364 -0.000002996

34 1 -0.000000127 -0.000000866 -0.000002740

35 1 -0.000000580 -0.000001625 0.000001464

36 1 0.000000386 -0.000000705 -0.000000655

37 1 0.000000882 0.000003652 0.000002452

38 1 0.000001070 0.000003417 -0.000000034

39 1 0.000001051 0.000001766 -0.000001173

40 1 0.000000701 0.000002847 -0.000000364

41 1 -0.000000577 -0.000002531 -0.000000259

42 1 -0.000001788 -0.000002880 -0.000001072

43 1 0.000000687 0.000001695 0.000001173

44 1 0.000000233 0.000000857 -0.000001305

45 1 0.000000065 -0.000000559 0.000000307

46 1 -0.000000716 -0.000000992 -0.000001238

-------------------------------------------------------------------

Zero-point correction= 0.339859 (Hartree/Particle)

Thermal correction to Energy= 0.366968

Thermal correction to Enthalpy= 0.367912

Thermal correction to Gibbs Free Energy= 0.275481

Sum of electronic and zero-point Energies= -1768.470712

Sum of electronic and thermal Energies= -1768.443603

Sum of electronic and thermal Enthalpies= -1768.442659

Sum of electronic and thermal Free Energies= -1768.535090

**Ts2**

---------------------------------------------------------------------

Center Atomic Atomic Coordinates (Angstroms)

Number Number Type X Y Z

---------------------------------------------------------------------

1 6 0 6.012839 -2.239176 -1.480506

2 6 0 7.341484 -2.539488 -1.766902

3 6 0 8.352006 -2.148582 -0.887491

4 6 0 8.018774 -1.457653 0.275043

5 6 0 6.686985 -1.155130 0.562854

6 6 0 5.673778 -1.542872 -0.314798

7 6 0 4.218030 -1.227523 -0.052193

8 8 0 2.814845 0.306997 2.808657

9 6 0 2.851522 -0.209145 1.712050

10 8 0 4.084413 -0.601846 1.219112

11 7 0 1.796612 -0.425094 0.886192

12 6 0 -0.468489 -0.821620 0.306121

13 6 0 0.443803 -0.153010 1.121337

14 6 0 -0.172602 0.585910 2.254323

15 6 0 -1.649177 0.331207 1.959156

16 7 0 -1.741114 -0.670995 0.963007

17 8 0 -2.586553 0.857590 2.514259

18 8 0 -0.257523 -1.536233 -0.687524

19 8 0 -3.023597 -0.384780 -0.040668

20 16 0 -3.087432 0.928952 -0.978507

21 8 0 -2.114118 1.920251 -0.515788

22 8 0 -3.094483 0.552957 -2.391511

23 6 0 -4.753636 1.515471 -0.594258

24 6 0 -5.076240 1.886411 0.710825

25 6 0 -6.355833 2.359606 0.977961

26 6 0 -7.320462 2.469029 -0.033384

27 6 0 -6.970255 2.080802 -1.328838

28 6 0 -5.692316 1.601152 -1.616006

29 6 0 -8.697497 3.007561 0.274061

30 1 0 5.231326 -2.548339 -2.168500

31 1 0 7.587636 -3.079799 -2.674694

32 1 0 9.387954 -2.382397 -1.107878

33 1 0 8.797029 -1.150783 0.965762

34 1 0 6.422953 -0.620827 1.466054

35 1 0 3.633577 -2.156409 -0.084356

36 1 0 1.908969 -0.929091 0.014096

37 1 0 0.049766 0.170979 3.245124

38 1 0 0.043128 1.660831 2.305550

39 1 0 -4.341283 1.779258 1.500503

40 1 0 -6.612039 2.642983 1.994513

41 1 0 -7.704155 2.148615 -2.126198

42 1 0 -5.418439 1.292509 -2.617004

43 1 0 -9.390889 2.821004 -0.549353

44 1 0 -9.112300 2.548604 1.176335

45 1 0 -8.668495 4.089530 0.444954

46 1 0 3.841896 -0.563898 -0.842010

---------------------------------------------------------------------

Zero-point correction= 0.336360 (Hartree/Particle)

Thermal correction to Energy= 0.363247

Thermal correction to Enthalpy= 0.364192

Thermal correction to Gibbs Free Energy= 0.272831

Sum of electronic and zero-point Energies= -1768.543608

Sum of electronic and thermal Energies= -1768.516721

Sum of electronic and thermal Enthalpies= -1768.515777

Sum of electronic and thermal Free Energies= -1768.607137

**Ts3 enol form**

-------------------------------------------------------------------

Center Atomic Forces (Hartrees/Bohr)

Number Number X Y Z

-------------------------------------------------------------------

1 6 0.000000572 0.000000482 -0.000003458

2 6 0.000000572 -0.000000461 -0.000001391

3 6 0.000000275 -0.000001489 -0.000000043

4 6 0.000000127 -0.000001728 0.000001643

5 6 -0.000000893 -0.000002709 0.000003906

6 6 0.000002628 -0.000001222 0.000000806

7 6 -0.000003759 -0.000001482 0.000002899

8 8 0.000007000 0.000006157 0.000004322

9 6 0.000003889 0.000000009 -0.000000386

10 8 0.000004235 0.000003400 0.000002933

11 7 -0.000006071 -0.000000572 -0.000003501

12 6 -0.000001193 -0.000003301 0.000001941

13 6 -0.000000803 0.000004554 -0.000005674

14 6 -0.000006666 -0.000000918 -0.000005319

15 6 0.000001179 0.000001997 0.000000069

16 7 -0.000000312 0.000000028 -0.000000238

17 8 0.000000429 0.000001903 -0.000001236

18 8 -0.000000202 -0.000000818 0.000005571

19 1 0.000001109 0.000000179 -0.000002951

20 1 0.000001099 -0.000000059 -0.000002917

21 1 0.000000240 -0.000001429 0.000000111

22 1 -0.000000620 -0.000002514 0.000003186

23 1 -0.000000302 -0.000001684 0.000002246

24 1 0.000000525 -0.000001401 -0.000000660

25 1 0.000001648 -0.000005326 -0.000001756

26 1 -0.000004035 0.000005054 0.000001837

27 1 -0.000000909 0.000004878 -0.000001201

28 1 0.000000242 -0.000001527 -0.000000740

-------------------------------------------------------------------

Zero-point correction= 0.205771 (Hartree/Particle)

Thermal correction to Energy= 0.220400

Thermal correction to Enthalpy= 0.221344

Thermal correction to Gibbs Free Energy= 0.160784

Sum of electronic and zero-point Energies= -873.409863

Sum of electronic and thermal Energies= -873.395235

Sum of electronic and thermal Enthalpies= -873.394291

Sum of electronic and thermal Free Energies= -873.454851

**Ts4 Keto form**

-------------------------------------------------------------------

Center Atomic Forces (Hartrees/Bohr)

Number Number X Y Z

-------------------------------------------------------------------

1 6 0.000000913 -0.000001609 -0.000003176

2 6 0.000002734 -0.000001939 0.000000233

3 6 0.000001826 -0.000002523 0.000000427

4 6 0.000000861 -0.000000915 -0.000001638

5 6 -0.000000781 0.000001270 -0.000002457

6 6 -0.000000487 -0.000001159 -0.000002748

7 6 0.000000226 -0.000002085 -0.000002878

8 8 -0.000004827 0.000004298 -0.000000961

9 6 -0.000002005 0.000000986 -0.000001648

10 8 -0.000001312 0.000007932 -0.000003112

11 7 -0.000001706 0.000000429 0.000000808

12 6 0.000001015 -0.000000472 0.000001598

13 6 0.000001566 0.000002006 0.000001556

14 6 -0.000000828 -0.000001089 0.000000397

15 6 -0.000000932 0.000001565 0.000006154

16 7 0.000000622 -0.000000596 0.000003369

17 8 -0.000000022 0.000000916 0.000007452

18 8 0.000000769 -0.000001615 0.000000825

19 1 0.000002326 -0.000002771 -0.000001683

20 1 0.000003324 -0.000003474 0.000000257

21 1 0.000002445 -0.000002686 0.000000846

22 1 0.000000399 -0.000000394 -0.000000492

23 1 -0.000000716 0.000001162 -0.000002369

24 1 -0.000000823 0.000001730 -0.000003957

25 1 0.000001314 -0.000002483 0.000002487

26 1 -0.000001750 0.000002165 0.000003525

27 1 -0.000003165 0.000001372 0.000003030

28 1 -0.000000988 -0.000000020 -0.000005846

-------------------------------------------------------------------

Zero-point correction= 0.202684 (Hartree/Particle)

Thermal correction to Energy= 0.217912

Thermal correction to Enthalpy= 0.218856

Thermal correction to Gibbs Free Energy= 0.156680

Sum of electronic and zero-point Energies= -873.284600

Sum of electronic and thermal Energies= -873.269372

Sum of electronic and thermal Enthalpies= -873.268428

Sum of electronic and thermal Free Energies= -873.330604

**References**

Aburabie, J., Emwas, A.H. andPeinemann, K.V. (2019). Silane-Crosslinked Asymmetric Polythiosemicarbazide Membranes for Organic Solvent Nanofiltration. Macromol. Mater. Eng., 304(1). doi: 10.1002/mame.201800551

Adamu, S., Atiqullah, M., Malaibari, Z.O., Al-Harthi, M.A., Emwas, A.H.M. and Ul-Hamid, A. (2016). Metallocene-catalyzed ethylene-alpha-olefin isomeric copolymerization: A perspective from hydrodynamic boundary layer mass transfer and design of MAO anion. J. Taiwan Inst. Chem. Eng., 60, 92-105. doi: 10.1016/j.jtice.2015.10.031

Alahmari, F., Davaasuren, B., Emwas, A.H., Costa, P.M.F.J. and Rothenberger, A. (2019). Tris(ethylenediamine)nickel(II) thio-hydroxogermanate monohydrate: Synthesis, crystal structure,H- 1 NMR, EPR, optical and magnetic properties*.* Inorganica Chim. Acta, 488, 145-151. doi: 10.1016/j.ica.2019.01.019.

Ali, M., Shawa, D.R., Zhang, L., Haroon, M.F., Narita, Y., Emwas, A.H., Saikaly, P.E. and Okabeb, S. (2018). Aggregation ability of three phylogenetically distant anammox bacterial species*.* Water Res., 143, 10-18. doi: 10.1016/j.watres.2018.06.007.

Ali, S., Badshah, G., Da Ros Montes D’Oca, C., Campos, F.R., Nagata, N., Khan, A., Maria de Santos, F.C. and Barison, A. (2020). High-Resolution Magic Angle Spinning (HR-MAS) NMR-Based Fingerprints Determination in the Medicinal PlantBerberis laurina. Molecules, 25(16), 3647. doi: 10.3390/molecules25163647.

Aljuhani, M.A., Zhang, Z., Barman, S., El Eter, M., Failvene, L., Ould-Chikh, S., Guan, E., Abou-Hamad, E., Emwas, A.H., Pelletier, J.D.A., Gates, B.C., Cavallo, L. and Basset, J.M.Aljuhani, M.A. (2019). Mechanistic Study of Hydroamination of Alkyne through Tantalum-Based Silica-Supported Surface Species. Acs Catalysis, 9(9), 8719-8725. doi: 10.1021/acscatal.9b02184.

de Graaf, R.A. and Behar, K.L. (2003). Quantitative H-1 NMR spectroscopy of blood plasma metabolites. Anal. Chem., 75(9), 2100-2104. doi: 10.1021/ac020782.

Atiqullah, M., Al-Harthi, M.A., Anantawaraskul, S. and Emwas, A.H. (2015). Ethylene homo- and copolymerization chain-transfers: A perspective from supported (nBuCp)(2)ZrCl2 catalyst active centre distribution. J. Chem. Sci., 127(4), 717-728. doi: 10.1007/s12039-015-0828-8.

Arda, A. and Jimenez-Barbero, J. (2018). The recognition of glycans by protein receptors. Insights from NMR spectroscopy. Chem.Comm., 54(38), 4761-4769. doi: 10.1039/C8CC01444B.

Arrabal-Campos, F.M., Aguilera-Saez, L.M.and Fernandez, I. (2019). Algebraic Reconstruction Technique for Diffusion NMR Experiments. Application to the Molecular Weight Prediction of Polymers. J. Phys. Chem. A, 123(4), 943-950. doi: 10.1021/acs.jpca.8b08584.

Asghar, S., Shahzadi, T., Alazmi, M., Gao, X., Emwas, A.H., Saleem, R.S.Z., Batool, F. and Chotana, G.A. (2018). Iridium-Catalyzed Regioselective Borylation of Substituted Biaryls. Synthesis-Stuttgart, 50(11), 2211-2220. doi: 10.1055/s-0036-1591968.

Mattar, S.M., Emwas, A.H.and Calhoun, L.A. (2004). Spectroscopic studies of the intermediates in the conversion of 1,4,11,12-tetrahydro-9,10-anthraquinone to 9,10-anthraquinone by reaction with oxygen under basic conditions. J. Phys. Chem. A, 108(52), 11545-11553. doi: 10.1021/jp040280v.

Davaasuren, B., Emwas, A.H. and Rothenberger, A. (2017). MAu2GeS4-Chalcogel (M = Co, Ni): Heterogeneous Intra- and Intermolecular Hydroamination Catalysts. Inorg. Chem., 56(16), 9609-9616. doi: 10.1021/acs.inorgchem.7b01099.

Atiqullah, M., Anantawaraskul, S., Emwas, A.H., Al-Harthi, M.A., Hussain, I., Ul-Hamid, A. and Hossaen, A. (2013). Effects of Supported ((BuCp)-Bu-n)(2)ZrCl2 Catalyst Active-Center Distribution on Ethylene-1-Hexene Copolymer Backbone Heterogeneity and Thermal Behaviors. Ind. Eng. Chem. Res., 52, 27, 9359–9373. doi: 10.1021/ie4005139.

Emwas, A.H., Szczepski, K., Poulson, B.G., Chandra, K., McKay, R.T., Dhahri, M., Alahmari, F., Jaremko, Ł., Lachowicz, J.I. and Jaremko, M. (2020) NMR as a "Gold Standard" Method in Drug Design and Discovery. Molecules, 25(20), 4597. doi: 10.3390/molecules25204597.

Quesne, le W. J. and Young, G. T. (1952). Amino-acids and peptides. Part VI. Synthesis of L-aspartyl peptides from carbobenzyloxy-L-aspartic anhydride. J. Chem. Soc., 1952, 24-28. doi: 10.1039/JR9520000024.

Stefanowicz, P., Jaremko, Ł., Jaremko, M. and Lis, T. (2005). Crystal-state studies on p-toluenesulfonates of N-oxyimides-a possible structural basis of serine proteases inhibition. New J. Chem., 30, 258-265. doi: 10.1039/B513741A.

Wunsch, E. (1974). in Methoden der Organischen Chemie. Synthese von Peptiden, Georg Thieme Verlag Stuttgart, p. 220.

Cal, M., Jaremko, M., Jaremko, Ł. and Stefanowicz, P. (2013). Solid phase synthesis of peptide hydroxamic acids on poly(ethylene glycol)-based support, J. Pept. Sci., 19, 9-15. doi: 10.1002/psc.2466

Silverstein, R. M., Webster, F. X. and Kiemle, D. J. (2005) Spectrometric identification of organic compounds, John Wiley & Sons, California.
